# Supplementary material for: Cost-effectiveness of cell salvage and donor blood transfusion during caesarean section: results from a randomised controlled trial
Source: BMJ Open. 2019 Feb 9;9(2):e022352. doi: 10.1136/bmjopen-2018-022352 (PMC6398743; doi:10.1136/bmjopen-2018-022352)
Supplement: Author's manuscript [file bmjopen-2018-022352.draft_revisions.pdf]

BMJ Open is committed to open peer review. As part of this commitment we make the peer review history of every article we publish publicly available.

When an article is published we post the peer reviewers' comments and the authors' responses online. We also post the versions of the paper that were used during peer review. These are the versions that the peer review comments apply to.

The versions of the paper that follow are the versions that were submitted during the peer review process. They are not the versions of record or the final published versions. They should not be cited or distributed as the published version of this manuscript.

BMJ Open is an open access journal and the full, final, typeset and author-corrected version of record of the manuscript is available on our site with no access controls, subscription charges or pay-per-view fees (http://bmjopen.bmj.com).

If you have any questions on BMJ Open's open peer review process please email editorial.bmjopen@bmj.com

Cost-effectiveness of cell salvage and donor blood transfusion during caesarean section: results from a randomised controlled trial

Journal:	*BMJ Open*
Manuscript ID	bmjopen-2018-022352
Article Type:	Research
Date Submitted by the Author:	27-Feb-2018
Complete List of Authors:	McLoughlin, Carol; University of Birmingham, Health Economics Unit<br>Roberts, Tracy; University of Birmingham, Health Economics Unit<br>Jackson, Louise; University of Birmingham, Health Economics Unit<br>Moore, Philip; Birmingham Women's Hospital, Selwyn Crawford Department of Anaesthetics<br>Wilson, Matthew; University of Sheffield, School of Health and Related Research (ScHARR)<br>Hooper, Richard; Queen Mary University of London, Centre for Primary Care & Public Health<br>Allard, Shubha; NHS Blood and Transplant and Barts Health NHS Trust<br>Wrench, Ian; Sheffield Teaching Hospitals NHS Foundation Trust Department of Anaesthesia<br>Beresford, Lee; Queen Mary University of London, Centre for Primary Care & Public Health<br>Geoghan, James; Birmingham Women's Hospital, Selwyn Crawford Department of Anaesthetics<br>Daniels, Jane; University of Nottingham, Nottingham Clinical Trials Unit<br>Catling, Sue ; Singleton Hospital, Department of Anaesthetics<br>Clark, Vicki; Royal Infirmary of Edinburgh, Simpson Centre for Reproductive Health<br>Ayuk, Paul; Royal Victoria Infirmary, Women's Services<br>Robson, Stephen; NIHR CRN North East and North Cumbria<br>Gao, Fang; University of Birmingham, Institute of Inflammation and Ageing, College of Medical and Dental Sciences; Heart of England NHS Foundation Trust, Academic Department of Anaesthesia, Critical Care, Pain and Resuscitation<br>Hogg, Matthew; Royal London Hospital, Barts Health NHS Trust<br>Lanz, Doris; Queen Mary University of London, Centre for Psychiatry<br>Dodds, Julie; Barts and The London School of Medicine and Dentistry, Women's Health Research Unit<br>Khan, Khalid; Queen Mary, University of London, Centre for Primary Care and Public Health
Keywords:	HEALTH ECONOMICS, Clinical trials < THERAPEUTICS, Maternal medicine < OBSTETRICS, Blood bank & transfusion medicine < HAEMATOLOGY

SCHOLARONE™
Manuscripts

Cost-effectiveness of cell salvage and donor blood transfusion during caesarean section: results from a randomised controlled trial

C McLoughlin[1], TE Roberts[1*], L Jackson[1], Philip AS Moore,[2] Matthew J Wilson,[3] Richard Hooper,[4] Shubha Allard,[5] Ian Wrench,[6] Lee Beresford,[4] James Geoghegan,[2] Jane P Daniels,[7] Sue Catling,[8] Vicki A Clark,[9] Paul Ayuk,[10] Stephen Robson,[11] Fang Gao-Smith,[12] Matthew Hogg,[13] Doris Lanz,[14] Julie Dodds,[14] Khalid S Khan[14] on behalf of the SALVO study group

[1] Health Economics Unit, University of Birmingham, B15 2TT UK

[2] Birmingham Women's Hospital, Birmingham, B15 2TH UK

[3] School of Health and Related Research (ScHARR), University of Sheffield, S1 4DA UK

[4] Pragmatic Clinical Trials Unit, Centre for Primary Care and Public Health, Queen Mary University of London, E1 2AB UK

[5] NHS Blood and Transplant, London, NW9 5BG UK

[6] Sheffield Teaching Hospitals NHS Foundation Trust, Sheffield, S10 2JF UK

[7] Nottingham Clinical Trials Unit, University of Nottingham, NG7 2UH UK

[8] Singleton Hospital, Swansea, SA2 8QA UK

[9] Simpson Centre for Reproductive Health, Royal Infirmary, Edinburgh, EH16 4SA UK

[10] Royal Victoria Infirmary, Newcastle-upon-Tyne, NE1 4LP UK

[11] Institute of Cellular Medicine, Newcastle University, NE2 4HH UK

[12] Perioperative, Critical Care and Trauma Trials Group, University of Birmingham, B15 2WB UK

[13] Royal London Hospital, Barts Health NHS Trust, London, E1 1BB UK

For peer review only - http://bmjopen.bmj.com/site/about/guidelines.xhtml

[14] Women's Health Research Unit, Barts and the London School of Medicine and Dentistry, Queen Mary University of London, E1 2AB UK

*Corresponding Author

Email: t.e.roberts@bham.ac.uk

For peer review only

For peer review only - http://bmjopen.bmj.com/site/about/guidelines.xhtml

ABSTRACT

Objectives: To evaluate the cost-effectiveness of routine use of cell salvage during caesarean section in mothers at risk of haemorrhage compared with current standard of care.

Design: Model-based cost-effectiveness evaluation alongside a multicentre randomised controlled trial. Three main analyses were carried out on the trial data: (1) based on the intention to treat principle; (2) based on the per protocol principle; (3) only participants who underwent an emergency caesarean section.

Setting: 26 obstetric units in the UK.

Participants: 3028 women at risk of haemorrhage recruited between June 2013 and April 2016.

Interventions: Cell salvage (intervention) versus routine care without salvage (control).

Primary outcome measures: Cost-effectiveness based on incremental cost per donor blood transfusion avoided.

Results: In Analysis 1 the mean difference in total costs between cell salvage and standard care was £83. The estimated incremental cost-effectiveness ratio (ICER) was £8,110 per donor blood transfusion avoided. For Analysis 2 the mean difference in total costs was £92 and the ICER was £8,252. In Analysis 3 the mean difference in total costs was £55 and the ICER was £13,713 per donor blood transfusion avoided. This ICER is driven by the increased probability that these patients would require a higher level of post-operative care and additional surgeries. The results of these analyses were shown to be robust for the majority of deterministic sensitivity analyses.

Conclusions: The results of the economic evaluation suggest that while routine cell salvage is a marginally more effective strategy than standard care in avoiding a donor blood

For peer review only - http://bmjopen.bmj.com/site/about/guidelines.xhtml

transfusion there is uncertainty in relation to whether it is a less or more costly strategy. The lack of long term data on the health and quality of life of patients in both arms of the trial means that further research is needed to fully understand the cost implications of both strategies.

Trial registration number: Current Controlled Trials ISRCTN66118656

STRENGTHS AND LIMITATIONS OF THIS STUDY

- Study strengths of this model-based economic evaluation include that it was based on a rigorously conducted RCT and it benefitted from significant clinical and statistical input throughout its design and development.
- The analyses were conducted from a healthcare perspective, and the cost and outcome data measures incorporated into the model were collected prospectively during the RCT using forms filled out during the pre-intra- and postoperative phases and at the time of discharge from hospital.
- A limitation of the study is that not all potential outcomes have been included because of the limited time scale in the model and the lack of long-term data.
- A further limitation of the evaluation is that outcomes were expressed in terms of clinical effectiveness rather than in terms of a standard unit of benefit such as the QALY (quality-adjusted life year).

Funding: Funding for this study was provided by the Health Technology Assessment Programme of the National Institute for Health Research. Project number 10/57/32.

For peer review only - http://bmjopen.bmj.com/site/about/guidelines.xhtml

Disclaimer: The views and opinions expressed herein are those of the authors and do not necessarily reflect those of the Health Technology Assessment programme, the National Institute for Health Research, the National Health Service, or the Department of Health.

Competing interests: None declared.

Data sharing statement: No additional data available.

Word Count: 3,226 words (not including title page, abstract, references, tables, figures)

Reporting statement: Consolidated Health Economic Evaluation Reporting Standards (CHEERS) statement provided.

For peer review only

INTRODUCTION

Excessive blood loss (haemorrhage) in childbirth is a life threatening condition which is an important cause of maternal death[1], emergency hysterectomy[2], and maternal critical care admission[3] among women undergoing a caesarean section[4]. The treatment for major haemorrhage includes donor blood transfusion. However, red cell concentrates used in donor transfusion are a scarce, nationally pooled resource in demand simultaneously by many clinical services[5]. Furthermore such transfusions carry risks for recipients as a result of incompatibility or infection[6]. Consequently there is a recognised need to make caesarean safer while at the same time promoting the use of transfusion alternatives harnessing the patient's own reserves where feasible[7, 8].

Intraoperative cell salvage (IOCS), which collects, processes, and returns the woman's own blood lost during surgery, is increasingly being deployed during caesareans. It has been shown to reduce the need for donor blood transfusions in a wide spectrum of surgical disciplines[9, 10].

This study presents the results of a model based economic evaluation which compared the relative cost and cost-effectiveness of routine cell salvage with standard transfusion with donor blood in caesarean section. This study is part a wider NIHR-HTA Randomised Controlled Trial exploring the effectiveness and cost-effectiveness of the use of cell salvage compared to donor blood transfusion during caesarean section[11]. Primary data on costs and resource use were collected prospectively alongside the trial and the principal outcome for the economic evaluation was the cost per donor blood transfusion avoided.

For peer review only - http://bmjopen.bmj.com/site/about/guidelines.xhtml

For peer review only

METHODS

Full details of the randomised controlled trial (RCT) are reported elsewhere[11]. Briefly, the study was performed in 26 obstetric units. The sample comprised women who were admitted to the labour ward for delivery by emergency or elective caesarean section, with an identifiable increased risk of haemorrhage. Participating women were randomised to either caesarean section with cell salvage (intervention group), or to caesarean section without cell salvage (control group) for which transfusion of donor blood was carried out according to local guidelines. The primary outcome for the randomised controlled trial was the proportion of women receiving donor blood transfusion due to haemorrhage. For the economic analysis a decision analytic model was deemed the most suitable method of presenting the alternative pathways and collating the data for analysis. The economic evaluation took the form of a cost-effectiveness analysis from the perspective of the health care provider based on the principal clinical outcome of the trial i.e. the proportion of women receiving donor blood transfusion due to haemorrhage. Given the objectives of the trial and the duration of follow up, outcomes beyond the trial endpoint were not considered plausible or relevant in the model based analysis.

Model structure

A decision tree model was developed in TreeAge Pro 2016 (TreeAge Software, Inc., Williamstown, MA, USA) to represent the alternative strategies. The pathways of the model (see Figure 1) represent, as far as possible, the clinical procedures carried out in the study.

Clinical data used in the model

For peer review only - http://bmjopen.bmj.com/site/about/guidelines.xhtml

The model combines the probability of a woman following a particular path and the associated costs. Probabilities, detailed in Table 1, were obtained from the trial and attached to each pathway.

Resource use and costs

The resource use for both arms of the trial was estimated by evaluating the individual components of these procedures (bottom-up costing). Unit cost data was then attached to the resource use. Data were collected on all major NHS resource use for each patient using the trial case report forms.

For the analysis, intraoperative and postoperative resource use data were obtained from the SALVO trial. The main resource use monitored included: equipment and disposables required for the cell salvage procedure; additional staff called into theatre solely for the purposes of cell salvage; drugs used in the caesarean section procedure; length and type of hospital inpatient stay including any additional treatment required attributed to the caesarean procedure; the use of donor blood transfusion and salvaged blood transfusion to deal with haemorrhage and its consequences.

Intra-operative costs were estimated for each item to arrive at a mean cost per caesarean procedure for each treatment pathway in the model. To estimate the cost of a caesarean section procedure some costs were calculated at the patient level e.g. swab washing and some at the procedural level e.g. drugs used in the caesarean section procedure (see Table 2). Postoperative costs were estimated for each item based on their occurrence in each brand of the model to arrive at a mean cost per patient for each branch (see Table 3).

For peer review only - http://bmjopen.bmj.com/site/about/guidelines.xhtml

Analysis

Given the objectives of the trial and the duration of follow up, a within trial economic analysis was carried out. The analysis took the perspective of the NHS following current recommendations from NICE[12]. The main economic analysis was a cost-effectiveness analysis (CEA) with results expressed as cost per donor transfusion avoided.

We carried out three main analyses on the trial data. Analysis 1, referred to as the base-case, was based on the intention-to-treat (ITT) principle. In this method, patients are compared within the treatment groups to which they were originally randomised irrespective of the treatment received[13]. This method of analysis allows the estimates to follow real-life scenarios in which patients may not always receive the planned treatment. ITT is recommended to ensure that the benefits of a given intervention are not exaggerated [13]. Analysis 2 was based on the treatment actually received by patients irrespective of randomisation (the 'per protocol' (PP) analysis). Within the SALVO trial patients were randomised to cell salvage or standard care in a 1:1 ratio. However, because some clinicians managing women in the control arm had access to a cell salvage machine, it was possible that women in the control arm could receive cell salvage in place of a donor blood transfusion. A PP analysis was carried out to look at the effect of treatment received on the outcome estimates. Therefore, in Analysis 2 all patients who received cell salvage were compared with those who received standard care, irrespective of the treatment to which they were randomised. Analysis 3 considered only patients who underwent an emergency caesarean section. This analysis was considered necessary as the SALVO trial found that numerically, there was a greater reduction in the rate of transfusion within the emergency

For peer review only - http://bmjopen.bmj.com/site/about/guidelines.xhtml

patient group compared to the elective patient group. This analysis followed the same methodology as the ITT and PP analyses. Deterministic and probabilistic sensitivity analyses (PSAs) were carried out for each analysis to explore the effects of the inherent uncertainty in parameter estimates on model results (summarised in Table 4).

Patient and public involvement

The UK National Childbirth Trust collaborated in the project by providing patient and public input through involvement in trial design and protocol development. Prior to the SALVO trial, a survey was conducted among women who received cell salvage, showing that they perceived the interventions as reassuring, safe, and preferable to donor blood transfusion. A patient representative was a member of the trial steering committee to provide oversight and advice regarding recruitment, dissemination, and general trial management. We are planning to disseminate finding to participants in the form of a newsletter following primary publication of these results.

RESULTS

The results of the ITT analysis (Analysis 1) suggest that routine cell salvage is more costly than standard care with the average cost per patient estimated at £1,244. The Incremental Cost-Effectiveness Ratio (ICER) representing the additional cost of routine cell salvage during caesarean section in women at risk of haemorrhage compared to standard care for these women was estimated to be approximately £8,110 per donor blood transfusion avoided. The overall result, based on the PP analysis (Analysis 2), was an ICER of £8,252 per transfusion avoided for cell salvage compared to standard care. The emergency caesarean analysis (Analysis 3) resulted in an ICER of £13,713 per transfusion avoided. See Table 5.

For peer review only - http://bmjopen.bmj.com/site/about/guidelines.xhtml

The results of the corresponding PSAs are presented using cost-effectiveness acceptability curves (CEACs) to graphically represent uncertainty in the appropriate threshold cost-effectiveness value. The CEAC for the ITT analysis (Figure 2) shows that the probability that routine cell salvage is cost-effective increases as the willingness to pay for a donor blood transfusion avoided increases. If the maximum willingness to pay for a donor blood transfusion avoided was £50,000, the probability of routine cell salvage being cost-effective would be 62%. For Analysis 2 (the PP analysis - Figure 3) the probability that routine cell salvage was cost-effective at a willingness to pay of £50,000, would be 63%. The CEAC for the emergency caesarean analysis (Analysis 3 - Figure 4) shows that the probability that cell salvage is cost-effective remains between 47% and 55% as the willingness to pay for a donor blood transfusion avoided increases.

Deterministic sensitivity analysis

A number of deterministic sensitivity analyses were conducted in each analysis (see Table 4):

(i) The main analyses used costs for consumables based on a particular model of the cell saver machine. To assess the difference that variation in these estimates would make, the unit costs were replaced with unit costs obtained from the NICE costing statement for blood transfusion[14]. This had a marginal impact on the ICER in each analysis. The impact of the inclusion of acquisition costs for a cell salvage machine was also explored and found this had only a minimal impact on the ICER in each analysis. In the trial, 202 centres used a cell salvage machine that did not require consumables. The impact of including costs for the consumables used by this

For peer review only - http://bmjopen.bmj.com/site/about/guidelines.xhtml

machine, where the machine is only set up for processing in patients having blood returned and where swab washing is not conducted, resulted in an ICER of £1,022 in Analysis 1 (ITT), £1,184 in Analysis 2 (PP analysis). In Analysis 3,(emergency caesarean analysis) there was a dominant result in that cell salvage was considered less costly and more effective compared to standard care.

(ii) The base case analyses (Analysis 1) used the mean length of time additional staff were present in theatre in each arm solely for the purposes of cell salvage. In the sensitivity analysis the cost of additional staff was removed. This reduced the ICER to £7,065 in the ITT analysis, £7,210 in the PP analysis, and £10,932 in the emergency caesarean analysis.

(iii) To facilitate robust evaluation in cost-effectiveness analyses relating to donor blood, a comprehensive estimate for the cost of a unit of donor blood is required. The NHS Blood and Transplant Authority (NHSBTA) have valued the cost of RBC to be £120 per unit based on direct costs to the healthcare services[15]. However, there is significant uncertainty surrounding this figure. We conducted a study (submitted for publication) parallel to the SALVO trial that aimed to dissect the current price of blood. We explored what elements are contributing to the current cost of blood and what elements are missing. Our study concluded that the current costing approach of assuming there will always be an adequate supply of donor blood must be replaced with including provisions for the continued shrinking of the donor pool and the impact that future shocks to the blood supply system could have. This sensitivity analysis assessed the difference that variation in the estimated cost of blood made to the overall cost-effectiveness of routine cell salvage. Raising the cost of a three unit transfusion of RBC to £1,500 reduced the ICER by £974 in both the ITT and PP

For peer review only

analysis and it reduced the ICER by over £1,000 in the emergency caesarean analysis. Threshold analysis showed that for routine cell salvage to be considered cost-effective, the cost of an RBC transfusion would have to be £8,637 in the ITT analysis, £8,778 in the PP analysis, and £13,186 in the emergency caesarean analysis.

DISCUSSION

Principal findings

The results of this economic evaluation suggest that routine cell salvage is more costly than standard care with the average cost per patient estimated at £1,327. The Incremental Cost Effectiveness Ratio (ICER) of this strategy compared with standard care is approximately £8,110 per donor blood transfusion avoided. This means that using cell salvage costs an additional £8,110 for every unit of donor blood avoided compared to standard care. The Probabilistic Sensitivity Analysis (PSA) suggests that at an arbitrary willingness to pay threshold of £50,000, the probability of routine cell salvage being cost-effective is 62%. These results suggest that cell salvage is not likely to be considered a cost-effective alternative to donor blood whilst donor blood is provided freely by individuals and supply is not typically restricted even though it is not a costless provision to the NHS.

The results of this analysis were shown to be robust for the majority of deterministic sensitivity analyses with one exception; using a cell salvage machine that required different consumables to those included in the main base case analyses where the machine is only set up for processing in patients having blood returned and where swab washing is not conducted resulted in a significant effect on the ICER, reducing it to £1,022 per donor blood transfusion avoided.

For peer review only - http://bmjopen.bmj.com/site/about/guidelines.xhtml

A per protocol analysis produced an ICER of £8,252 per transfusion avoided, but this result should be considered with caution as the population in this analysis is a subset of the ITT population who completed the study without any major protocol violations[13]. In clinical practice uptake of cell salvage is unlikely to be "per-protocol". In a third analysis, looking at emergency caesareans only, cell salvage appears to be more effective than standard care for avoiding a donor blood transfusion but the resulting ICER of £13,713 is driven by the increased probability that these patients will require a higher level of post-operative care and additional surgeries.

Strengths and limitations of the study

The strength of this model based economic evaluation is that it was based on a rigorously conducted Randomised Controlled Trial (RCT). The cost and outcome data measures that were incorporated into the model were collected prospectively during the RCT using forms filled out at the pre-, intra-, and post-operative phase and at the time of discharge from hospital. In addition the economic evaluation benefitted from significant clinical and statistical input throughout its design and development. All assumptions used in the model were agreed with the trial team before the analysis was carried out and without knowledge of how these assumptions would affect the results.

In terms of limitations, it was not possible to account for long-term implications relating to maternal fetal exposure as data relating to this wasn't available from the trial. In addition, information relating to the clinical status and care of the infant was not included in the analysis. A further limitation of the study is that outcomes were expressed in terms of

For peer review only - http://bmjopen.bmj.com/site/about/guidelines.xhtml

clinical effectiveness rather than in terms of a standard unit of benefit such as the quality-adjusted life-year (QALY). Finally, the use of platelets and other blood products has not been included in the study. However, the results of the sensitivity and threshold analyses demonstrated that including these costs would not have impacted on the cost-effectiveness results.

Strengths and weaknesses in relation to other studies

To date, there has only been one, small, RCT looking at the elective use of cell salvage at caesarean section[16] and this study did not include an economic element. A Cochrane review of cell salvage in adult elective surgery assessed the clinical and cost-effectiveness of cell salvage and other autologous transfusion strategies in elective surgery[10]. It suggested that cell salvage may be an 'effective and cost-effective alternative to the allogeneic blood transfusion strategy'. However, no obstetric papers were identified for this review.

Meaning of the study

The results of the economic evaluation suggest that while routine cell salvage is a marginally more effective strategy than standard care in avoiding a donor blood transfusion there is uncertainty in relation to whether it is a less or more costly strategy. Under the conditions reported here, for a high income country (HIC) such as the UK, where donor blood is typically available, cell salvage is unlikely to be considered a cost-effective alternative to the provision of donor blood by the health service. However, in lower and middle income countries (LMIC) where the provision of a safe and secure blood supply may be more challenging the relative cost-effectiveness may be very different. In addition, the lack of long term data on the health and quality of life of patients in both arms of the trial means that

For peer review only - http://bmjopen.bmj.com/site/about/guidelines.xhtml

further research is needed to fully understand the cost implications of both strategies. For example, latent infection such as hepatitis may result in chronic liver disease within 20 or more years of incident of infection [17, 18] which has obvious long term cost implications for the health care provider.

Unanswered questions and future research

The current study has used data from a large, multicentre randomised trial which demonstrated modest evidence that routine use of cell salvage during caesarean section reduced the need for donor blood transfusion. The main cause of uncertainty relates to the cost implications of adopting the routine use of cell salvage. Future studies should explore the long-term health and economic impacts associated with both transfusion strategies. Also, evidence on the preferences of women needs to be considered. For example hospitals may wish to have the option of cell salvage available for Jehovah witness patients where there is no option to use donor blood. In countries where safe donor supply cannot be guaranteed the use of cell salvage might have very different implications which need to be explored.

Finally, the issue of donor blood as a scarce resource needs to be considered. As things currently stand demand for donor blood is increasing while on the other hand enhanced safety measures are limiting the donor pool [19, 20]. The impact of further restrictions on supply could create shortages under current usage patterns, and donor blood substitutes such as cell salvage play a potential role in helping to re-establish a demand-supply balance [21, 22]. Not considered in this study is that fact that transfusion with cell salvage can always exist. While there is an expectation that donor blood will always be there when

For peer review only - http://bmjopen.bmj.com/site/about/guidelines.xhtml

needed, transfusion using donor blood simply cannot be guaranteed. In such a scenario, where the option of donor blood is limited or not available, the routine use of cell salvage would be dominant (less costly and more effective) compared to standard care thus making provision for the availability of the technology likely to be extremely important.

For peer review only

For peer review only - http://bmjopen.bmj.com/site/about/guidelines.xhtml

362 **Acknowledgements:** The study group would like to that local clinical midwifery, theatre, obstetric, and anaesthetic teams for their support, as well as members of the trial steering and data monitoring committees, patient and public representatives, staff at the Women's Health Research Unit and Pragmatic Clinical Trials Unit at Queen Mary University of London, and all the women who agreed to participate in the SALVO trial.

368 **Contributors:** CML undertook the economic analysis, interpreted the results, and drafted the manuscript with input from all authors. TR contributed to the trial design, developed the economic analysis plan, supervised the economic analysis and revised the paper. LJ contributed to the economic analysis plan and revised the paper. PM contributed to the trial design, provided clinical advice and revised the paper. MW contributed to the trial design and revised the paper. RH contributed to the trial design, developed the trial statistical plan and revised the paper. SA contributed to the trial design and revised the paper. IW contributed to the trial design and revised the paper. LB performed the trial statistical analysis and revised the paper. JG, JD, SC, AV, PA, SR, FGS and MH contributed to the trial design and revised the paper. DL supervised the general management of the trial, supervised data collection and revised the paper. JD supervised the running of the trial and revised the paper. KK was the chief investigator for the trial, contributed to the trial design, managed the trial and revised the paper.

For peer review only - http://bmjopen.bmj.com/site/about/guidelines.xhtml

REFERENCES

1. Centre for Maternal and Child Enquiries (CMAEC), *Saving Mothers' Lives: reviewing maternal deaths to make motherhood safer: 2006–08. The Eighth Report on Confidential Enquiries into Maternal Deaths in the United Kingdom.* BJOG: an International Journal of Obstetrics and Gynaecology, 2011. **118**(Suppl. 1).
2. Bodelon, C., et al., *Factors associated with peripartum hysterectomy.* Obstetrics and gynecology, 2009. **114**(1): p. 115.
3. Intensive Care National Audit & Research Centre (ICNARC), *Female Admissions (Aged 16-50 years) to Adult, General Critical Care Units in England, Wales and Northern Ireland Reported as 'Currently Pregnant' or 'Recently Pregnant'*. 2013.
4. Bateman, B.T., et al., *The epidemiology of postpartum hemorrhage in a large, nationwide sample of deliveries.* Anesthesia & Analgesia, 2010. **110**(5): p. 1368-1373.
5. NHS Blood and Transplant.*Saving and Improving Lives. Strategic Plan 2015-20.*2015 [accessed:September 18 2017] Available from: https://nhsbtdbe.blob.core.windows.net/umbraco-assets-corp/1593/strategic_plan_2015_20.pdf
6. Catling, S., *Intraoperative cell salvage in obstetrics.* Clinical Risk, 2008. **14**(1): p. 14-17.
7. Mehra, T., et al., *Implementation of a patient blood management monitoring and feedback program significantly reduces transfusions and costs.* Transfusion, 2015. **55**(12): p. 2807-2815.
8. Shander, A., J. Isbister, and H. Gombotz, *Patient blood management: the global view.* Transfusion, 2016. **56**(S1).
9. Carless, P.A., et al., *Cell salvage for minimising perioperative allogeneic blood transfusion.* Cochrane Database Syst Rev, 2010. **4**(4).
10. Davies, L., et al., *Cost-effectiveness of cell salvage and alternative methods of minimising perioperative allogeneic blood transfusion: a systematic review and economic model.* Health Technology Assessment, 2006. **10**(44).
11. Khan, K.S., et al., *A randomised controlled trial and economic evaluation of intraoperative cell salvage during caesarean section in women at risk of haemorrhage: the SALVO (cell SALVage in Obstetrics) trial.* Health Technol Assess 2018. **22**(2).
12. National Institute for Health and Care Excellence (NICE), *Guide to the methods of technology appraisal*. 2013, Springer.
13. Gupta, S.K., *Intention-to-treat concept: a review.* Perspectives in clinical research, 2011. **2**(3): p. 109.
14. National Institute for Health and Care Excellence (NICE), *Costing Statement: Blood Transfusion. Implementing the NICE Guideline on Blood Transfusion (NG24).* 2015.
15. NHS Blood and Transplant.*Blood and Components Price List 2016/2017.*2016 [accessed:September 18 2017] Available from: http://hospital.blood.co.uk/media/28230/component-price-list-2016-2017.pdf
16. Rainaldi, M., et al., *Blood salvage during caesarean section.* British journal of anaesthesia, 1998. **80**(2): p. 195-198.
17. Seeff, L.B., et al., *Long-term mortality after transfusion-associated non-A, non-B hepatitis.* New England Journal of Medicine, 1992. **327**(27): p. 1906-1911.
18. Shalmani, H.M., M. Ranjbar, and A.H.M. Alizadeh, *Recommendations for prevention and control of hepatitis C virus (HCV) infection and HCV-related chronic disease.* J Liver, 2013. **3**(147): p. 2167-0889.1000147.
19. Busch, M.P., S.H. Kleinman, and G.J. Nemo, *Current and emerging infectious risks of blood transfusions.* Jama, 2003. **289**(8): p. 959-962.
20. Williamson, L.M. and D.V. Devine, *Challenges in the management of the blood supply.* The Lancet, 2013. **381**(9880): p. 1866-1875.

For peer review only - http://bmjopen.bmj.com/site/about/guidelines.xhtml

21. Spahn, D.R. and L.T. Goodnough, *Alternatives to blood transfusion.* The Lancet, 2013. **381**(9880): p. 1855-1865.
22. Dixon, S., et al., *Economic analysis of the implementation of autologous transfusion technologies throughout England.* International journal of technology assessment in health care, 2005. **21**(2): p. 234-239.
23. NHS Supply Chain.*NHS Supply Chain Catalogue*.2015 [accessed:August 2016] Available from: https://my.supplychain.nhs.uk/catalogue
24. UK Cell Salvage Action Group, *ICS Technical Fact Sheet: Swab Washing*. 2015.
25. Committee, J.F.*British National Formulary*.2016 [accessed:August 2016] Available from: https://bnf.nice.org.uk/
26. Curtis L, *Unit Costs of Health and Social Care 2015*. 2015, University of Kent: PSSRU.
27. Qureshi, H., et al., *BCSH guideline for the use of anti-D immunoglobulin for the prevention of haemolytic disease of the fetus and newborn.* Transfusion Medicine, 2014. **24**(1): p. 8-20.
28. Department of Health.*NHS Reference Costs 2014/2015*.2015 [accessed:August 2016] Available from: https://www.gov.uk/government/uploads/system/uploads/attachment_data/file/477919/2014-15_Reference_costs_publication.pdf
29. NHS National Tariff Payment System.*2016/17 National Prices*.2016 [accessed:August 2016] Available from: www.gov.uk/government/publications/national-tariff-payment-system-2014-to-2015
30. Tinegate, H., et al., *Guideline on the investigation and management of acute transfusion reactions Prepared by the BCSH Blood Transfusion Task Force.* British journal of haematology, 2012. **159**(2): p. 143-153.

For peer review only - http://bmjopen.bmj.com/site/about/guidelines.xhtml

TABLES

Table 1 Probabilities used in the model

	Trial data	Probability	Distribution
**Cell salvage intended**			
Cell salvage intended → allocated treatment received (machine is on)	1432 / 1498	0.96	Beta
Allocated treatment received → salvaged blood returned	726/1432	0.51	Beta
Allocated treatment received → salvaged blood not returned	703/1432	0.49	Beta
Salvaged blood returned → donor blood transfusion given	22/726	0.03	Beta
Salvaged blood returned → donor blood transfusion not given	704 /726	0.97	Beta
Salvaged blood not returned → donor blood transfusion given	9/703	0.01	Beta
Salvaged blood not returned → donor blood transfusion not given	697 / 703	0.99	Beta
Cell salvage intended → allocated treatment not received (machine is off)	66/1498	0.04	Beta
Allocated treatment not received → donor blood transfusion given	6/66	0.09	Beta
Allocated treatment not received → donor blood transfusion not given	60/66	0.91	Beta
**Standard care intended**			
Standard care intended → allocated treatment received (machine is off)	1434/1492	0.96	Beta
Allocated treatment received → donor blood transfusion given	47/1434	0.03	Beta
Allocated treatment received → donor blood transfusion not given	1387 / 1434	0.97	Beta
Standard care intended → allocated treatment not received (machine is on)	58/1492	0.04	Beta
Allocated treatment not received → salvaged blood returned	35/58	0.60	Beta
Allocated treatment not received → salvaged blood not returned	23/58	0.40	Beta
Salvaged blood returned → donor blood transfusion given	4/35	0.11	Beta
Salvaged blood returned → donor blood transfusion not given	31/35	0.89	Beta
Salvaged blood not returned → donor blood transfusion given	1/23	0.04	Beta
Salvaged blood not returned → donor blood transfusion not given	22/23	0.96	Beta

For peer review only

Table 2 Intraoperative resource use and costs

Item	Resource Use: Cell salvage (n=1498)	Resource Use: Control (n=1492)	Unit Cost	Mean cost per procedure: Cell salvage (n-=1498)	Mean cost per procedure: Control (n=1492)	Assumption / Working	Source
Running costs	1432	58	£6.14	£6.14	£6.14	Based on annual maintenance costs for Haemonetics Cell Saver 5 (Haemonetics UK Ltd) machine and estimated annual usage	UHB, (Mr Scott Hancock, University Hospitals Birmingham, 2016, personal communication); NICE costing statement blood transfusion (Nov 2015) [14]
Collection Set	1	1	£41.71	£41.71	£41.71	Based on the assumption that one collection set is used per procedure	NHS Supply Chain Catalogue Autotransfusion reservoir 3 l [23]
Processing Pack	1	1	£77	£77	£77	Based on the assumption that one processing pack is used per procedure	NHS Supply Chain Catalogue Intraoperative autologous blood system cell saver 5+ bowl set 125ml (Haemonetics UK Ltd) [23]
Leukocyte depletion filter	782	25	n/a	n/a	n/a	Cost not included in the analysis as leukocyte depletion filter included in the collection set for Haemonetics Cell Saver 5 machine (Haemonetics UK Ltd)	NHS Supply Chain Catalogue Autotransfusion reservoir 3 litre[23]
Additional sucker	598	29	£15.41	£6.43	£7.70	Mean cost based on the number of additional suckers used in each treatment arm / total number of patients who received cell salvage	NHS Supply Chain Catalogue Aspiration & anticoagulation line Cell Saver. (Haemonetics UK Ltd) £308.02 for 20[23]
Swab washing	781	21	£0.80	£0.44	£0.29	Mean cost based on the number of times swabs were washed in each treatment group/total number of patients who received cell salvage	ICS Factsheet 1 Swab Washing March 2015 [24], based on the cost of 1l of sodium chloride 0.9%, BNF[25]
Staff			£0.72 (min)	£11.57	£12.03	Based on the staff type most frequently called into theatre	Unit cost for hospital based nurse, band 5, PSSRU unit costs 2015 (costs include qualifications) [26]
Saline (l)	2	2	£0.80	£1.60	£1.60	Based on the assumption that 2 l of saline would be administered to all patients undergoing cell salvage prior to collection[14]	Based on the cost of 1l of 0.9% sodium chloride, BNF[25]
Heparin	2	2	£10.60	£21.20	£21.20	Based on the assumption that 60,000 IU of heparin	Based on the cost of 1-ml amp of heparin sodium

For peer review only

For peer review only - http://bmjopen.bmj.com/site/about/guidelines.xhtml

sodium (30,000 IU)						would be administered to all patients undergoing cell salvage prior to collection[14]	25,000 IU/ml and 1-ml amp of heparin sodium 5,000 IU/ml, BNF[25]
Anti-D (500 IU)	1	1	£33.75	£3.04	£3.04	Based on the assumption that all RhD-negative women delivering a RhD-positive baby receive at least 500 IU of anti-D[27]. Mean cost per procedure based on the probability of a woman requiring anti-D in each treatment arm (0.09)	Based on the cost of 500-unit vial of anti-D immunoglobulin, BNF[25]
Anti-D (1500 IU)	1	1	£58	£5.22	£5.22	Based on the assumption that women who receive cell salvage are offered 1500 IU of anti-D[27]. Mean cost per procedure based on the probability of a woman requiring anti-D in each treatment arm (0.09)	Based on the cost of 1,500-unit vial of anti-D immunoglobulin, BNF[25]
RBC transfusion (units)	3	3	First unit: £194 Subsequent units: £166	£520	£520	Based on the assumption that all units transfused in each treatment arm were RBC[14]	NICE costing statement for blood transfusion (November 2015)[14]. Unit cost for RBCs obtained from NHSBT 2016/17[15]

For peer review only

For peer review only - http://bmjopen.bmj.com/site/about/guidelines.xhtml

Table 3 Postoperative resource use and costs

	Resource Use			Mean cost per patient		
	Cell salvage	Control		Cell salvage	Control	
**Item**	(n=1498)	(n=1492)	**Unit Cost**	(n-=1498)	(n=1492)	**Source**
Inpatient stay (normal days)	3734.5	3852	£431.45	£1,074	£1,113	NHS reference costs 2014/15[28]. Weighted average unit cost for elective and non-elective inpatient bed days
Inpatient stay (HLC)	189.5	136	£539 - £848*	£78	£56	NHS reference costs 2014/15 [28] National tariff payment system 2016/17 [29]
Adverse events	3	0	n/a	n/a	n/a	Assumption that transfusion would be discontinued in the event of an adverse reaction based on BCSH guidelines [30]
Hospital transfer	2	2	£99	£0.13	£0.13	PSSRU 2015 [26]
Investigations	6	10	£94 - £138†	£0.42	£0.70	NHS reference costs 2014/15 [28]
Additional surgery	11	8	£399 - £2,991‡	£13	£9	NHS reference costs 2014/15 [28]
RBC transfusion (units)	3	3	First unit: £194 Subsequent units: £166	£13	£17	NICE costing statement for blood transfusion (November 2015). Unit cost for RBC obtained from NHSBT 2016/17 [15]
Total cost of postnatal care per patient				£1178.55	£1195.86	

* Range based on cost per day of care: Level 1 £539, Level 2 £674, Level 3 £848

† Range based on unit cost of a CT scan (£94) and an MRI scan (£138)

‡ Range based on unit cost of additional surgeries (less cost of days in hospital)

For peer review only - http://bmjopen.bmj.com/site/about/guidelines.xhtml

For peer review only

Table 4 Deterministic sensitivity analysis

	Original value		Revised value	Original result		
				Cost per donor blood transfusion avoided		
				**Analysis 1**	**Analysis 2**	**Analysis 3**
				£8110	£8252	£13,713
				**Revised result**		
**(i) Equipment and disposables required for the cell salvage procedure**						
Applying the cost of consumables (collection set + processing pack) used by NICE	£118.71		£119.75	£8205	£8346	£13,952
Including acquisition costs based on Haemonetics Cell Saver machine	-		£22.13	£10,114	£10,246	£18,805
	Cell salvage	Control				
Using a continuous transfusion cell saver where the machine is only set up for processing in patients having blood returned and swab washing is not conducted	£125.14	£126.40	£34.73	£1022	£1184	Dominates
**(ii) Staff**						
No additional member of staff being called into theatre solely for the purposes of cell salvage	Cell salvage £11.57	Control £12.03	£0	£7065	£7210	£10,932
**(iii) Donor blood**						
Variation in the estimate of the cost of a 3 unit RBC transfusion	£520		£750	£7886	£8028	£13,330
			£1000	£7636	£7778	£13,080
			£1250	£7386	£7528	£12,830
			£1500	£7136	£7278	£12,580
For routine cell salvage to be considered cost-effective in this model the price of a 3 unit RBC transfusion would have to be:				£8637	£8778	£13,186

For peer review only - http://bmjopen.bmj.com/site/about/guidelines.xhtml

474 **Table 5 Results**

475

Transfusion strategy	Trial data (n)	Average cost per patient (£)	Difference in costs (£)	Effectiveness (Donor blood transfusion avoided)	Incremental donor blood transfusion avoided	ICER per donor blood transfusion avoided (£)
**Analysis 1 (ITT analysis)**						
Standard care	1492	1244		0.965		
Cell salvage	1498	1327	83	0.975	0.010	8110
**Analysis 2 (PP analysis)**						
Standard care	1434	1238		0.967		
Cell salvage	1432	1330	92	0.978	0.011	8252
**Analysis 3 (Emergency caesarean analysis)**						
Standard care	808	1352		0.986		
Cell salvage	833	1407	55	0.990	0.004	13713

For peer review only

For peer review only - http://bmjopen.bmj.com/site/about/guidelines.xhtml

476

For peer review only

For peer review only - http://bmjopen.bmj.com/site/about/guidelines.xhtml

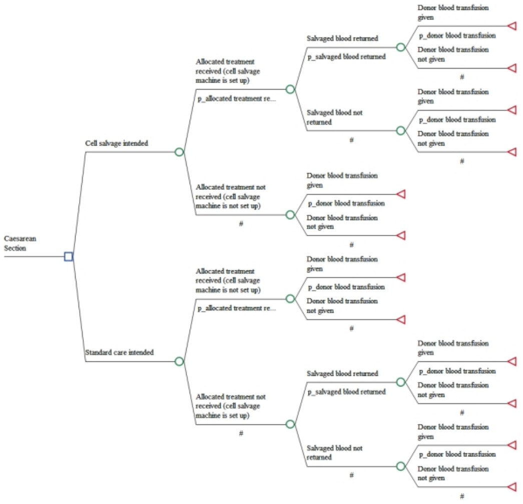

Decision tree structure

167x86mm (300 x 300 DPI)

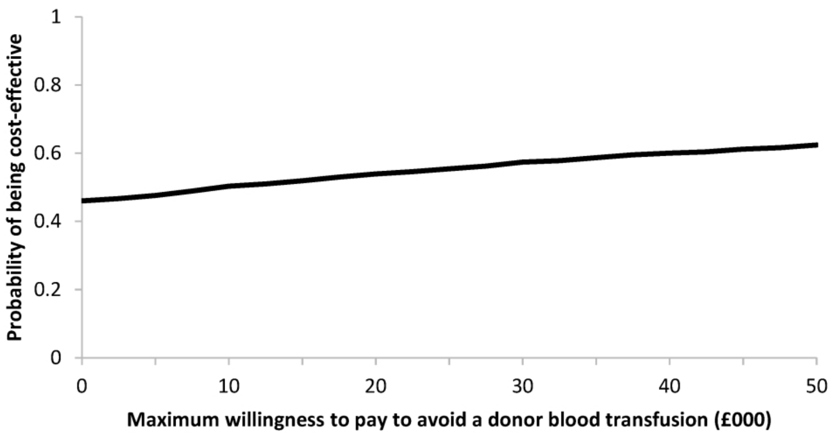

Cost-effectiveness acceptability curve for donor blood transfusion avoided (ITT)

297x210mm (300 x 300 DPI)

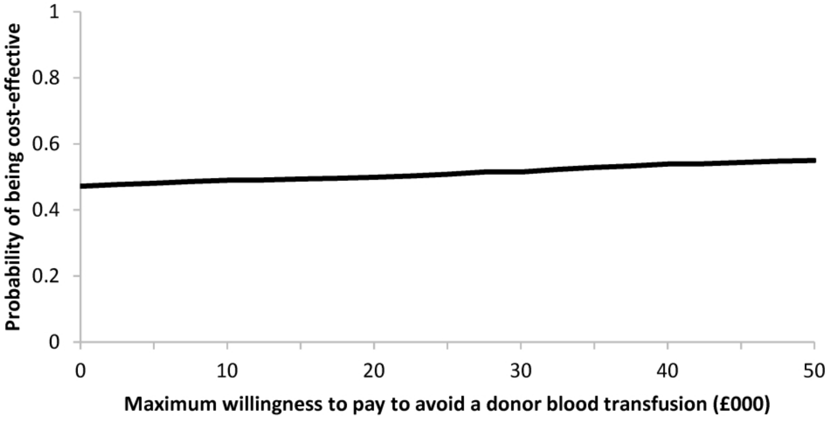

Cost-effectiveness acceptability curve for donor blood transfusion avoided (PP)

296x209mm (300 x 300 DPI)

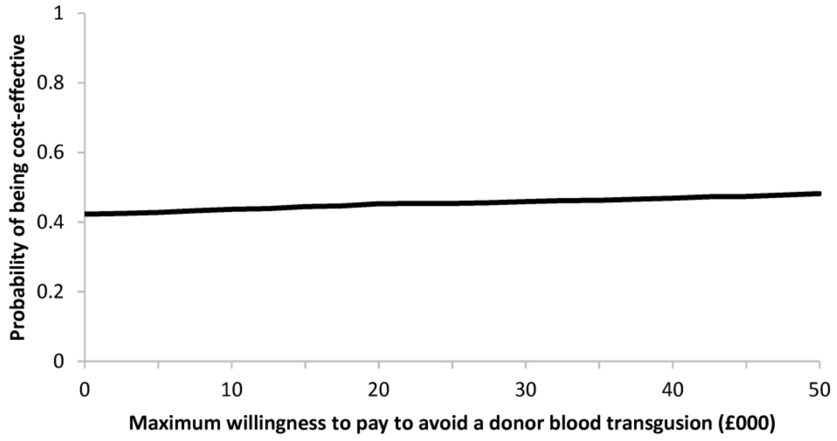

Cost-effectiveness acceptability curve for donor blood transfusion avoided (emergency only)

296x209mm (300 x 300 DPI)

The CHEERS Checklist is part of the CHEERS Statement. The CHEERS Statement has been endorsed and co-published by the following journals:

BJOG: An International Journal of Obstetrics and Gynaecology
BMC Medicine 2013; 11:80
BMJ 2013;346:f1049
Clinical Therapeutics 27 March 2013 (Article in Press DOI: 10.1016/j.clinthera.2013.03.003)
Cost Effectiveness and Resource Allocation 2013 11:6.
The European Journal of Health Economics 2013 Mar 26. [Epub ahead of print]
International Journal of Technology Assessment in Health Care
Journal of Medical Economics 2013 Mar 25. [Epub ahead of print]
Pharmacoeconomics 2013 Mar 26. [Epub ahead of print]
Value in Health 2013 March - April;16(2):e1-e5

CHEERS Checklist
Items to include when reporting economic evaluations of health interventions

Section/item	Item No	Recommendation	Reported on page No/ line No
**Title and abstract**			
Title	1	Identify the study as an economic evaluation or use more specific terms such as "cost-effectiveness analysis", and describe the interventions compared.	1/1
Abstract	2	Provide a structured summary of objectives, perspective, setting, methods (including study design and inputs), results (including base case and uncertainty analyses), and conclusions.	3/32
**Introduction**			
Background and objectives	3	Provide an explicit statement of the broader context for the study. Present the study question and its relevance for health policy or practice decisions.	6/92
**Methods**			
Target population and subgroups	4	Describe characteristics of the base case population and subgroups analysed, including why they were chosen.	7/118
Setting and location	5	State relevant aspects of the system(s) in which the decision(s) need(s) to be made.	7/118
Study perspective	6	Describe the perspective of the study and relate this to the costs being evaluated.	7/127
Comparators	7	Describe the interventions or strategies being compared and state why they were chosen.	7/121
Time horizon	8	State the time horizon(s) over which costs and consequences are being evaluated and say why appropriate.	7/130
Discount rate	9	Report the choice of discount rate(s) used for costs and	n/a

		outcomes and say why appropriate.	[illegible]
Choice of health outcomes	10	Describe what outcomes were used as the measure(s) of benefit in the evaluation and their relevance for the type of analysis performed.	7 / 128
Measurement of effectiveness	11a	*Single study-based estimates:* Describe fully the design features of the single effectiveness study and why the single study was a sufficient source of clinical effectiveness data.	n/a
	11b	*Synthesis-based estimates:* Describe fully the methods used for identification of included studies and synthesis of clinical effectiveness data.	n/a
Measurement and valuation of preference based outcomes	12	If applicable, describe the population and methods used to elicit preferences for outcomes.	n/a
Estimating resources and costs	13a	*Single study-based economic evaluation:* Describe approaches used to estimate resource use associated with the alternative interventions. Describe primary or secondary research methods for valuing each resource item in terms of its unit cost. Describe any adjustments made to approximate to opportunity costs.	n/a
	13b	*Model-based economic evaluation:* Describe approaches and data sources used to estimate resource use associated with model health states. Describe primary or secondary research methods for valuing each resource item in terms of its unit cost. Describe any adjustments made to approximate to opportunity costs.	7/133
Currency, price date, and conversion	14	Report the dates of the estimated resource quantities and unit costs. Describe methods for adjusting estimated unit costs to the year of reported costs if necessary. Describe methods for converting costs into a common currency base and the exchange rate.	8 / 143
Choice of model	15	Describe and give reasons for the specific type of decision-analytical model used. Providing a figure to show model structure is strongly recommended.	7/133
Assumptions	16	Describe all structural or other assumptions underpinning the decision-analytical model.	7/133
Analytical methods	17	Describe all analytical methods supporting the evaluation. This could include methods for dealing with skewed, missing, or censored data; extrapolation methods; methods for pooling data; approaches to validate or make adjustments (such as half cycle corrections) to a model; and methods for handling population heterogeneity and uncertainty.	9 / 164
**Results**			
Study parameters	18	Report the values, ranges, references, and, if used, probability distributions for all parameters. Report reasons or sources for distributions used to represent uncertainty where appropriate. Providing a table to show the input values is strongly recommended.	7/138

Incremental costs and outcomes	19	For each intervention, report mean values for the main categories of estimated costs and outcomes of interest, as well as mean differences between the comparator groups. If applicable, report incremental cost-effectiveness ratios.	10/202
Characterising uncertainty	20a	*Single study-based economic evaluation:* Describe the effects of sampling uncertainty for the estimated incremental cost and incremental effectiveness parameters, together with the impact of methodological assumptions (such as discount rate, study perspective).	n/a
	20b	*Model-based economic evaluation:* Describe the effects on the results of uncertainty for all input parameters, and uncertainty related to the structure of the model and assumptions.	11/224
Characterising heterogeneity	21	If applicable, report differences in costs, outcomes, or cost-effectiveness that can be explained by variations between subgroups of patients with different baseline characteristics or other observed variability in effects that are not reducible by more information.	n/a
**Discussion**			
Study findings, limitations, generalisability, and current knowledge	22	Summarise key study findings and describe how they support the conclusions reached. Discuss limitations and the generalisability of the findings and how the findings fit with current knowledge.	13/264
**Other**			
Source of funding	23	Describe how the study was funded and the role of the funder in the identification, design, conduct, and reporting of the analysis. Describe other non-monetary sources of support.	4/76
Conflicts of interest	24	Describe any potential for conflict of interest of study contributors in accordance with journal policy. In the absence of a journal policy, we recommend authors comply with International Committee of Medical Journal Editors recommendations.	5/83

For consistency, the CHEERS Statement checklist format is based on the format of the CONSORT statement checklist

The CHEERS Statement may be accessed by the publication links above.

The **ISPOR CHEERS Task Force Report** provides examples and further discussion of the 24-item CHEERS Checklist and the CHEERS Statement. It may be accessed via the *Value in Health* link or via the ISPOR Health Economic Evaluation Publication Guidelines – CHEERS: Good Reporting Practices webpage: http://www.ispor.org/TaskForces/EconomicPubGuidelines.asp

The citation for the CHEERS Task Force Report is:
Husereau D, Drummond M, Petrou S, et al. Consolidated health economic evaluation reporting standards (CHEERS)—Explanation and elaboration: A report of the ISPOR health economic evaluations publication guidelines good reporting practices task force. Value Health 2013;16:231-50.

BMJ Open

Cost-effectiveness of cell salvage and donor blood transfusion during caesarean section: results from a randomised controlled trial

Journal:	*BMJ Open*
Manuscript ID	bmjopen-2018-022352.R1
Article Type:	Research
Date Submitted by the Author:	06-Aug-2018
Complete List of Authors:	McLoughlin, Carol; University of Birmingham, Health Economics Unit<br>Roberts, Tracy; University of Birmingham, Health Economics Unit<br>Jackson, Louise; University of Birmingham, Health Economics Unit<br>Moore, Philip; Birmingham Women's Hospital, Selwyn Crawford Department of Anaesthetics<br>Wilson, Matthew; University of Sheffield, School of Health and Related Research (ScHARR)<br>Hooper, Richard; Queen Mary University of London, Centre for Primary Care & Public Health<br>Allard, Shubha; NHS Blood and Transplant and Barts Health NHS Trust<br>Wrench, Ian; Sheffield Teaching Hospitals NHS Foundation Trust Department of Anaesthesia<br>Beresford, Lee; Queen Mary University of London, Centre for Primary Care & Public Health<br>Geoghegan, James; Birmingham Women's Hospital, Selwyn Crawford Department of Anaesthetics<br>Daniels, Jane; University of Nottingham, Nottingham Clinical Trials Unit<br>Catling, Sue ; Singleton Hospital, Department of Anaesthetics<br>Clark, Vicki; Royal Infirmary of Edinburgh, Simpson Centre for Reproductive Health<br>Ayuk, Paul; Royal Victoria Infirmary, Women's Services<br>Robson, Stephen; NIHR CRN North East and North Cumbria<br>Gao, Fang; University of Birmingham, Institute of Inflammation and Ageing, College of Medical and Dental Sciences; Heart of England NHS Foundation Trust, Academic Department of Anaesthesia, Critical Care, Pain and Resuscitation<br>Hogg, Matthew; Royal London Hospital, Barts Health NHS Trust<br>Lanz, Doris; Queen Mary University of London, Centre for Psychiatry<br>Dodds, Julie; Barts and The London School of Medicine and Dentistry, Women's Health Research Unit<br>Khan, Khalid; Queen Mary, University of London, Centre for Primary Care and Public Health
**Primary Subject Heading**:	Health economics
Secondary Subject Heading:	Obstetrics and gynaecology, Health economics, Haematology (incl blood transfusion)
Keywords:	HEALTH ECONOMICS, Clinical trials < THERAPEUTICS, Maternal medicine

	< OBSTETRICS, Blood bank & transfusion medicine < HAEMATOLOGY, Health economics < HEALTH SERVICES ADMINISTRATION & MANAGEMENT

SCHOLARONE™
Manuscripts

Cost-effectiveness of cell salvage and donor blood transfusion during caesarean section: results from a randomised controlled trial

Carol McLoughlin[1], Tracy Roberts[1*], Louise J Jackson[1], Philip Moore,[2] Matthew Wilson,[3] Richard Hooper,[4] Shubha Allard,[5] Ian Wrench,[6] Lee Beresford,[4] James Geoghegan,[2] Jane Daniels,[7] Sue Catling,[8] Vicki A Clark,[9] Paul Ayuk,[10] Stephen Robson,[11] Fang Gao,[12] Matthew Hogg,[13] Doris Lanz,[14] Julie Dodds,[14] Khalid Khan[14] on behalf of the SALVO study group

[1] Health Economics Unit, University of Birmingham, B15 2TT UK

[2] Birmingham Women's Hospital, Birmingham, B15 2TH UK

[3] School of Health and Related Research (ScHARR), University of Sheffield, S1 4DA UK

[4] Pragmatic Clinical Trials Unit, Centre for Primary Care and Public Health, Queen Mary University of London, E1 2AB UK

[5] NHS Blood and Transplant, London, NW9 5BG UK

[6] Sheffield Teaching Hospitals NHS Foundation Trust, Sheffield, S10 2JF UK

[7] Nottingham Clinical Trials Unit, University of Nottingham, NG7 2UH UK

[8] Singleton Hospital, Swansea, SA2 8QA UK

[9] Simpson Centre for Reproductive Health, Royal Infirmary, Edinburgh, EH16 4SA UK

[10] Royal Victoria Infirmary, Newcastle-upon-Tyne, NE1 4LP UK

[11] Institute of Cellular Medicine, Newcastle University, NE2 4HH UK

[12] Perioperative, Critical Care and Trauma Trials Group, University of Birmingham, B15 2WB UK

[13] Royal London Hospital, Barts Health NHS Trust, London, E1 1BB UK

For peer review only

For peer review only - http://bmjopen.bmj.com/site/about/guidelines.xhtml

[14] Women's Health Research Unit, Barts and the London School of Medicine and Dentistry, Queen Mary University of London, E1 2AB UK

*Corresponding Author

Email: t.e.roberts@bham.ac.uk

For peer review only

For peer review only - http://bmjopen.bmj.com/site/about/guidelines.xhtml

ABSTRACT

Objectives: To evaluate the cost-effectiveness of routine use of cell salvage during caesarean section in mothers at risk of haemorrhage compared with current standard of care.

Design: Model-based cost-effectiveness evaluation alongside a multicentre RCT. Three main analyses were carried out on the trial data: (1) based on the intention-to-treat principle; (2) based on the per-protocol principle; (3) only participants who underwent an emergency caesarean section.

Setting: 26 obstetric units in the UK.

Participants: 3028 women at risk of haemorrhage recruited between June 2013 and April 2016.

Interventions: Cell salvage (intervention) versus routine care without salvage (control).

Primary outcome measures: Cost-effectiveness based on incremental cost per donor blood transfusion avoided.

Results: In the intention-to-treat analysis the mean difference in total costs between cell salvage and standard care was £83. The estimated incremental cost-effectiveness ratio (ICER) was £8,110 per donor blood transfusion avoided. For the per-protocol analysis the mean difference in total costs was £92 and the ICER was £8,252. In the emergency caesarean section analysis the mean difference in total costs was £55 and the ICER was £13,713 per donor blood transfusion avoided. This ICER is driven by the increased probability that these patients would require a higher level of post-operative care and additional surgeries. The results of these analyses were shown to be robust for the majority of deterministic sensitivity analyses.

For peer review only - http://bmjopen.bmj.com/site/about/guidelines.xhtml

Conclusions: The results of the economic evaluation suggest that while routine cell salvage is a marginally more effective strategy than standard care in avoiding a donor blood transfusion there is uncertainty in relation to whether it is a less or more costly strategy. The lack of long term data on the health and quality of life of patients in both arms of the trial means that further research is needed to fully understand the cost implications of both strategies.

Trial registration number: Current Controlled Trials ISRCTN66118656

STRENGTHS AND LIMITATIONS OF THIS STUDY

- Study strengths of this model-based economic evaluation include that it was based on a rigorously conducted RCT and it benefitted from significant clinical and statistical input throughout its design and development.
- The analyses were conducted from a healthcare perspective, and the cost and outcome data measures incorporated into the model were collected prospectively during the RCT using forms filled out during the pre-intra- and postoperative phases and at the time of discharge from hospital.
- A limitation of the study is that not all potential outcomes have been included because of the limited time scale in the model and the lack of long-term data.
- A further limitation of the evaluation is that outcomes were expressed in terms of clinical effectiveness rather than in terms of a standard unit of benefit such as the QALY (quality-adjusted life year).

Funding: Funding for this study was provided by the Health Technology Assessment Programme of the National Institute for Health Research. Project number 10/57/32.

For peer review only

For peer review only - http://bmjopen.bmj.com/site/about/guidelines.xhtml

Disclaimer: The views and opinions expressed herein are those of the authors and do not necessarily reflect those of the Health Technology Assessment programme, NIHR, NHS or the Department of Health.

Competing interests: None declared.

Data sharing statement: No additional data available.

Word Count: 3,442 words (not including title page, abstract, references, tables, figures)

Reporting statement: Consolidated Health Economic Evaluation Reporting Standards (CHEERS) statement provided.

For peer review only - http://bmjopen.bmj.com/site/about/guidelines.xhtml

INTRODUCTION

Excessive blood loss (haemorrhage) in childbirth is a life threatening condition which is an important cause of maternal death[1], emergency hysterectomy[2], and maternal critical care admission[3] among women undergoing a caesarean section[4]. The treatment for major haemorrhage includes donor blood transfusion. However, red cell concentrates used in donor transfusion are a scarce, nationally pooled resource in demand simultaneously by many clinical services[5]. Furthermore such transfusions carry risks for recipients as a result of incompatibility or infection[6]. Consequently there is a recognised need to make caesarean safer while at the same time promoting the use of transfusion alternatives harnessing the patient's own reserves where feasible[7, 8].

Intraoperative cell salvage (IOCS), which collects, processes, and returns the woman's own blood lost during surgery, is increasingly being deployed during caesareans. It has been shown to reduce the need for donor blood transfusions in a wide spectrum of surgical disciplines[9, 10].

This study presents the results of a model based economic evaluation which compared the relative cost and cost-effectiveness of routine cell salvage with standard transfusion with donor blood in caesarean section. This study is part a wider NIHR-HTA Randomised Controlled Trial exploring the effectiveness and cost-effectiveness of the use of cell salvage compared to donor blood transfusion during caesarean section[11]. Primary data on costs and resource use were collected prospectively alongside the trial and the principal outcome for the economic evaluation was the cost per donor blood transfusion avoided.

For peer review only - http://bmjopen.bmj.com/site/about/guidelines.xhtml

METHODS

Full details of the randomised controlled trial (RCT) are reported elsewhere[11]. Briefly, the study was performed in 26 obstetric units. The sample comprised women who were admitted to a participating labour ward and who fulfilled all of the following inclusion criteria: aged $\geq$ 16 years; able to provide informed consent; undergoing delivery by caesarean section with an identifiable increased risk of haemorrhage, defined as all emergency caesarean sections, where maternal or fetal compromise is suspected, and elective caesarean section for all indications other than maternal request or breech presentation. Participating women were randomised to either caesarean section with cell salvage (intervention group), or to caesarean section without cell salvage (control group) for which transfusion of donor blood was carried out according to local guidelines. The primary outcome for the randomised controlled trial was the proportion of women receiving donor blood transfusion due to haemorrhage. For the economic analysis a decision analytic model was deemed the most suitable method of presenting the alternative pathways and collating the data for analysis. The economic evaluation took the form of a cost-effectiveness analysis from the perspective of the health care provider based on the principal clinical outcome of the trial i.e. the proportion of women receiving donor blood transfusion due to haemorrhage. Given the objectives of the trial and the duration of follow up, outcomes beyond the trial endpoint were not considered plausible or relevant in the model based analysis.

For peer review only - http://bmjopen.bmj.com/site/about/guidelines.xhtml

Model structure

A decision tree model was developed in TreeAge Pro 2016 (TreeAge Software, Inc., Williamstown, MA, USA) to represent the alternative strategies. The pathways of the model (see Figure 1) represent, as far as possible, the clinical procedures carried out in the study.

Clinical data used in the model

The model combines the probability of a woman following a particular path and the associated costs. Probabilities, detailed in Table 1, were obtained from the trial and attached to each pathway.

Table 1 Probabilities used in the model

	Trial data	Probability	Distribution
**Cell salvage intended**			
Cell salvage intended → allocated treatment received (machine is on)	1432 / 1498	0.96	Beta
Allocated treatment received → salvaged blood returned	726/1432	0.51	Beta
Allocated treatment received → salvaged blood not returned	703/1432	0.49	Beta
Salvaged blood returned → donor blood transfusion given	22/726	0.03	Beta
Salvaged blood returned → donor blood transfusion not given	704 /726	0.97	Beta
Salvaged blood not returned → donor blood transfusion given	9/703	0.01	Beta
Salvaged blood not returned → donor blood transfusion not given	697 / 703	0.99	Beta
Cell salvage intended → allocated treatment not received (machine is off)	66/1498	0.04	Beta
Allocated treatment not received → donor blood transfusion given	6/66	0.09	Beta
Allocated treatment not received → donor blood transfusion not given	60/66	0.91	Beta
**Standard care intended**			
Standard care intended → allocated treatment received (machine is off)	1434/1492	0.96	Beta
Allocated treatment received → donor blood transfusion given	47/1434	0.03	Beta
Allocated treatment received → donor blood transfusion not given	1387 / 1434	0.97	Beta

For peer review only - http://bmjopen.bmj.com/site/about/guidelines.xhtml

Standard care intended → allocated treatment not received (machine is on)	58/1492	0.04	Beta
Allocated treatment not received → salvaged blood returned	35/58	0.60	Beta
Allocated treatment not received → salvaged blood not returned	23/58	0.40	Beta
Salvaged blood returned → donor blood transfusion given	4/35	0.11	Beta
Salvaged blood returned → donor blood transfusion not given	31/35	0.89	Beta
Salvaged blood not returned → donor blood transfusion given	1/23	0.04	Beta
Salvaged blood not returned → donor blood transfusion not given	22/23	0.96	Beta

Resource use and costs

The resource use for both arms of the trial was estimated by evaluating the individual components of these procedures (bottom-up costing). Unit cost data was then attached to the resource use. Data were collected on all major NHS resource use for each patient using the trial case report forms.

For the analysis, intraoperative and postoperative resource use data were obtained from the SALVO trial. The main resource use monitored included: equipment and disposables required for the cell salvage procedure; additional staff called into theatre solely for the purposes of cell salvage; drugs used in the caesarean section procedure; length and type of hospital inpatient stay including any additional treatment required attributed to the caesarean procedure; the use of donor blood transfusion and salvaged blood transfusion to deal with haemorrhage and its consequences.

Intra-operative costs were estimated for each item to arrive at a mean cost per caesarean procedure for each treatment pathway in the model. To estimate the cost of a caesarean section procedure some costs were calculated at the patient level e.g. swab washing and

For peer review only

For peer review only - http://bmjopen.bmj.com/site/about/guidelines.xhtml

some at the procedural level e.g. drugs used in the caesarean section procedure (see Table 2). Postoperative costs were estimated for each item based on their occurrence in each brand of the model to arrive at a mean cost per patient for each branch (see Table 3).

For peer review only

For peer review only - http://bmjopen.bmj.com/site/about/guidelines.xhtml

Table 2 Intraoperative resource use and costs

	Resource Use		Unit Cost	Mean cost per procedure			
**Item**	**Cell salvage** (n=1498)	**Control** (n=1492)		**Cell salvage** (n-=1498)	**Control** (n=1492)	**Assumption / Working**	**Source**
Running costs	1432	58	£6.14	£6.14	£6.14	Based on annual maintenance costs for Haemonetics Cell Saver 5 (Haemonetics UK Ltd) machine and estimated annual usage	UHB, (Mr Scott Hancock, University Hospitals Birmingham, 2016, personal communication); NICE costing statement blood transfusion (Nov 2015) [12]
Collection Set	1	1	£41.71	£41.71	£41.71	Based on the assumption that one collection set is used per procedure	NHS Supply Chain Catalogue Autotransfusion reservoir 3 l [13]
Processing Pack	1	1	£77	£77	£77	Based on the assumption that one processing pack is used per procedure	NHS Supply Chain Catalogue Intraoperative autologous blood system cell saver 5+ bowl set 125ml (Haemonetics UK Ltd) [13]
Leukocyte depletion filter	782	25	n/a	n/a	n/a	Cost not included in the analysis as leukocyte depletion filter included in the collection set for Haemonetics Cell Saver 5 machine (Haemonetics UK Ltd)	NHS Supply Chain Catalogue Autotransfusion reservoir 3 litre[13]
Additional sucker	598	29	£15.41	£6.43	£7.70	Mean cost based on the number of additional suckers used in each treatment arm / total number of patients who received cell salvage	NHS Supply Chain Catalogue Aspiration & anticoagulation line Cell Saver. (Haemonetics UK Ltd) £308.02 for 20[13]
Swab washing	781	21	£0.80	£0.44	£0.29	Mean cost based on the number of times swabs were washed in each treatment group/total number of patients who received cell salvage	ICS Factsheet 1 Swab Washing March 2015 [14], based on the cost of 1l of sodium chloride 0.9%, BNF[15]
Staff			£0.72 (min)	£11.57	£12.03	Based on the staff type most frequently called into theatre solely for the purposes of cell salvage	Unit cost for hospital based nurse, band 5, PSSRU unit costs 2015 (costs include qualifications) [16]
Saline (l)	2	2	£0.80	£1.60	£1.60	Based on the assumption that 2 l of saline would be administered to all patients undergoing cell salvage prior to collection[12]	Based on the cost of 1l of 0.9% sodium chloride, BNF[15]
Heparin	2	2	£10.60	£21.20	£21.20	Based on the assumption that 60,000 IU of heparin	Based on the cost of 1-ml amp of heparin sodium

For peer review only

For peer review only - http://bmjopen.bmj.com/site/about/guidelines.xhtml

sodium (30,000 IU)						would be administered to all patients undergoing cell salvage prior to collection[12]	25,000 IU/ml and 1-ml amp of heparin sodium 5,000 IU/ml, BNF[15]
Anti-D (500 IU)	1	1	£33.75	£3.04	£3.04	Based on the assumption that all RhD-negative women delivering a RhD-positive baby receive at least 500 IU of anti-D[17]. Mean cost per procedure based on the probability of a woman requiring anti-D in each treatment arm (0.09)	Based on the cost of 500-unit vial of anti-D immunoglobulin, BNF[15]
Anti-D (1500 IU)	1	1	£58	£5.22	£5.22	Based on the assumption that women who receive cell salvage are offered 1500 IU of anti-D[17]. Mean cost per procedure based on the probability of a woman requiring anti-D in each treatment arm (0.09)	Based on the cost of 1,500-unit vial of anti-D immunoglobulin, BNF[15]
RBC transfusion (units)	3	3	First unit: £194 Subsequent units: £166	£520	£520	Based on the assumption that all units transfused in each treatment arm were RBC[12]	NICE costing statement for blood transfusion (November 2015)[12]. Unit cost for RBCs obtained from NHSBT 2016/17[18]

For peer review only

For peer review only - http://bmjopen.bmj.com/site/about/guidelines.xhtml

Table 3 Postoperative resource use and costs

	Resource Use			Mean cost per patient		
	Cell salvage	Control		Cell salvage	Control	
**Item**	(n=1498)	(n=1492)	**Unit Cost**	(n-=1498)	(n=1492)	**Source**
Inpatient stay (normal days)	3734.5	3852	£431.45	£1,074	£1,113	NHS reference costs 2014/15[19]. Weighted average unit cost for elective and non-elective inpatient bed days
Inpatient stay (HLC)	189.5	136	£539 - £848*	£78	£56	NHS reference costs 2014/15 [19] National tariff payment system 2016/17 [20]
Adverse events	3	0	n/a	n/a	n/a	Assumption that transfusion would be discontinued in the event of an adverse reaction based on BCSH guidelines [21]
Hospital transfer	2	2	£99	£0.13	£0.13	PSSRU 2015 [16]
Investigations	6	10	£94 - £138†	£0.42	£0.70	NHS reference costs 2014/15 [19]
Additional surgery	11	8	£399 - £2,991‡	£13	£9	NHS reference costs 2014/15 [19]
RBC transfusion (units)	3	3	First unit: £194 Subsequent units: £166	£13	£17	NICE costing statement for blood transfusion (November 2015). Unit cost for RBC obtained from NHSBT 2016/17 [18]
Total cost of postnatal care per patient				£1178.55	£1195.86	

* Range based on cost per day of care: Level 1 £539, Level 2 £674, Level 3 £848

† Range based on unit cost of a CT scan (£94) and an MRI scan (£138)

‡ Range based on unit cost of additional surgeries (less cost of days in hospital)

For peer review only

For peer review only - http://bmjopen.bmj.com/site/about/guidelines.xhtml

ANALYSIS

Given the objectives of the trial and the duration of follow up, a within trial economic analysis was carried out. The analysis took the perspective of the NHS and PSS following current recommendations from NICE[22]. The main economic analysis was a cost-effectiveness analysis (CEA) with results expressed as cost per donor transfusion avoided.

We carried out three main analyses on the trial data. The first analysis (Analysis ITT), referred to as the base-case, was based on the intention-to-treat (ITT) principle. In this method, patients are compared within the treatment groups to which they were originally randomised irrespective of the treatment received [23]. This method of analysis allows the estimates to follow real-life scenarios in which patients may not always receive the planned treatment. ITT is recommended to ensure that the benefits of a given intervention are not exaggerated [23]. The second analysis (Analysis PP) was based on the treatment actually received by patients irrespective of randomisation (the 'per protocol' (PP) analysis). Within the SALVO trial, equal numbers of patients were randomised to either cell salvage or standard care. However, because some clinicians managing women in the control arm had access to a cell salvage machine, it was possible that women in the control arm could receive cell salvage in place of a donor blood transfusion. A PP analysis was carried out to look at the effect of treatment received on the outcome estimates. Therefore, in Analysis PP all patients who received cell salvage were compared with those who received standard care, irrespective of the treatment to which they were randomised. The third analysis (Analysis ECD) considered only patients who underwent an emergency caesarean section. This analysis was considered necessary as the SALVO trial found that numerically, there was a greater reduction in the rate of transfusion within the emergency patient group compared

For peer review only - http://bmjopen.bmj.com/site/about/guidelines.xhtml

to the elective patient group. This analysis followed the same methodology as the ITT and PP analyses. Deterministic and probabilistic sensitivity analyses (PSAs) were carried out for each analysis to explore the effects of the inherent uncertainty in parameter estimates on model results.

Patient and public involvement

The UK National Childbirth Trust collaborated in the project by providing patient and public input through involvement in trial design and protocol development. Prior to the SALVO trial, a survey was conducted among women who received cell salvage, showing that they perceived the interventions as reassuring, safe, ad preferable to donor blood transfusion. A patient representative was a member of the trial steering committee to provide oversight and advice regarding recruitment, dissemination, and general trial management. We are planning to disseminate findings to participants in the form of a newsletter following primary publication of these results.

RESULTS

The results of the ITT analysis (Analysis ITT) suggest that routine cell salvage is more costly than standard care with the mean difference in total costs per patient estimated at £83. The Incremental Cost-Effectiveness Ratio (ICER) representing the additional cost of routine cell salvage during caesarean section in women at risk of haemorrhage compared to standard care for these women was estimated to be approximately £8,110 per donor blood transfusion avoided. The overall result, based on the PP analysis (Analysis PP), was an ICER of £8,252 per transfusion avoided for cell salvage compared to standard care. The

For peer review only - http://bmjopen.bmj.com/site/about/guidelines.xhtml

emergency caesarean analysis (Analysis ECD) resulted in an ICER of £13,713 per transfusion avoided. See Table 4.

The results of the corresponding PSAs are presented using cost-effectiveness acceptability curves (CEACs) to graphically represent uncertainty in the appropriate threshold cost-effectiveness value. The CEAC for the ITT analysis (Figure 2) shows that the probability that routine cell salvage is cost-effective increases as the willingness to pay for a donor blood transfusion avoided increases. If the maximum willingness to pay for a donor blood transfusion avoided was £50,000, the probability of routine cell salvage being cost-effective would be 62%. For Analysis 2 (the PP analysis - Figure 2) the probability that routine cell salvage was cost-effective at a willingness to pay of £50,000, would be 63%. The CEAC for the emergency caesarean analysis (Analysis 3 - Figure 2) shows that the probability that cell salvage is cost-effective remains between 47% and 55% as the willingness to pay for a donor blood transfusion avoided increases.

For peer review only

For peer review only - http://bmjopen.bmj.com/site/about/guidelines.xhtml

Table 4 Results

Transfusion strategy	Average cost per patient (£)	Difference in costs (£)	Effectiveness (Donor blood transfusion avoided)	Incremental donor blood transfusion avoided	ICER per donor blood transfusion avoided (£)
**Analysis 1 (ITT analysis)**					
Standard care	1244		0.965		
Cell salvage	1327	83	0.975	0.010	8110
**Analysis 2 (PP analysis)**					
Standard care	1238		0.967		
Cell salvage	1330	92	0.978	0.011	8252
**Analysis 3 (Emergency caesarean analysis)**					
Standard care	1352		0.986		
Cell salvage	1407	55	0.990	0.004	13713

For peer review only

Deterministic sensitivity analysis

A number of deterministic sensitivity analyses were conducted in each analysis (see Table 5):

(i) The main analyses used costs for consumables based on a particular model of the cell saver machine. To assess the difference that variation in these estimates would make, the unit costs were replaced with unit costs obtained from the NICE costing statement for blood transfusion[12]. This had a marginal impact on the ICER in each analysis. The impact of the inclusion of acquisition costs for a cell salvage machine was also explored and found this had only a minimal impact on the ICER in each analysis. In the trial, 202 cases used a cell salvage machine that required consumables for collection only, even where the blood was not processed. The impact of including costs for the consumables used by this machine, where the machine is only set up for processing in patients having blood returned and where swab washing is not conducted, resulted in an ICER of £1,022 in Analysis ITT, £1,184 in Analysis PP. In Analysis ECD there was a dominant result in that cell salvage was considered less costly and more effective compared to standard care.

(ii) The base case analyses (Analysis ITT) used the mean length of time additional staff were present in theatre in each arm solely for the purposes of cell salvage. In the sensitivity analysis the cost of additional staff was removed. This reduced the ICER to £7,065 in Analysis ITT, £7,210 in Analysis PP, and £10,932 in Analysis ECD.

(iii) To facilitate robust evaluation in cost-effectiveness analyses relating to donor blood, a comprehensive estimate for the cost of a unit of donor blood is required. The NHS Blood and Transplant Authority (NHSBTA) have valued the cost of RBC to be £120 per unit based on direct costs to the healthcare services[18]. However, there is

For peer review only - http://bmjopen.bmj.com/site/about/guidelines.xhtml

significant uncertainty surrounding this figure. We conducted a study (submitted for publication) parallel to the SALVO trial that aimed to dissect the current price of blood. We explored what elements are contributing to the current cost of blood and what elements are missing. Our study concluded that the current costing approach of assuming there will always be an adequate supply of donor blood must be replaced with including provisions for the continued shrinking of the donor pool and the impact that future shocks to the blood supply system could have. This sensitivity analysis assessed the difference that variation in the estimated cost of blood made to the overall cost-effectiveness of routine cell salvage. Raising the cost of a three unit transfusion of RBC to a hypothetical cost of £1,500 reduced the ICER by £974 in both the ITT and PP analysis and it reduced the ICER by over £1,000 in the emergency caesarean analysis. Threshold analysis showed that for routine cell salvage to be considered cost-effective, the cost of an RBC transfusion would have to be £8,637 in Analysis ITT, £8,778 in Analysis PP, and £13,186 in Analysis ECD.

For peer review only - http://bmjopen.bmj.com/site/about/guidelines.xhtml

Table 5 Deterministic sensitivity analysis

	Original value	Revised value	Original result		
			Cost per donor blood transfusion avoided		
			**Analysis 1**	**Analysis 2**	**Analysis 3**
			£8110	£8252	£13,713
				**Revised result**	
**(i) Equipment and disposables required for the cell salvage procedure**					
Applying the cost of consumables (collection set + processing pack) used by NICE	£118.71	£119.75	£8205	£8346	£13,952
Including acquisition costs based on Haemonetics Cell Saver machine	-	£22.13	£10,114	£10,246	£18,805
Using a continuous transfusion cell saver where the machine is only set up for processing in patients having blood returned and swab washing is not conducted	Cell salvage £125.14; Control £126.40	£34.73	£1022	£1184	Dominates
**(ii) Staff**					
No additional member of staff being called into theatre solely for the purposes of cell salvage	Cell salvage £11.57; Control £12.03	£0	£7065	£7210	£10,932
**(iii) Donor blood**					
Variation in the estimate of the cost of a 3 unit RBC transfusion	£520	£750	£7886	£8028	£13,330
		£1000	£7636	£7778	£13,080
		£1250	£7386	£7528	£12,830
		£1500	£7136	£7278	£12,580
For routine cell salvage to be considered cost-effective in this model the price of a 3 unit RBC transfusion would have to be:			£8637	£8778	£13,186

For peer review only

For peer review only - http://bmjopen.bmj.com/site/about/guidelines.xhtml

DISCUSSION

Principal findings

The results of this economic evaluation suggest that routine cell salvage is more costly than standard care with the average cost per patient estimated at £1,327. The Incremental Cost Effectiveness Ratio (ICER) of this strategy compared with standard care is approximately £8,110 per donor blood transfusion avoided. This means that using cell salvage costs an additional £8,110 for every unit of donor blood avoided compared to standard care. The Probabilistic Sensitivity Analysis (PSA) suggests that at an arbitrary willingness to pay threshold of £50,000, the probability of routine cell salvage being cost-effective is 62%. Based on the results of this trial, in a UK setting the results suggest that cell salvage is not likely to be considered a cost-effective alternative to donor blood whilst donor blood is provided freely by individuals and supply is not typically restricted even though it is not a costless provision to the NHS.

The results of this analysis were shown to be robust for the majority of deterministic sensitivity analyses with one exception; using a cell salvage machine that required different consumables to those included in the main base case analyses where the machine is only set up for processing in patients having blood returned and where swab washing is not conducted resulted in a significant effect on the ICER, reducing it to £1,022 per donor blood transfusion avoided.

A per protocol analysis produced an ICER of £8,252 per transfusion avoided, but this result should be considered with caution as the population in this analysis is a subset of the ITT population who completed the study without any major protocol violations[23]. In clinical

For peer review only - http://bmjopen.bmj.com/site/about/guidelines.xhtml

practice uptake of cell salvage is unlikely to be "per-protocol". In a third analysis, looking at emergency caesareans only, cell salvage appears to be more effective than standard care for avoiding a donor blood transfusion but the resulting ICER of £13,713 is driven by the increased probability that these patients will require a higher level of post-operative care and additional surgeries.

Strengths and limitations of the study

The strength of this model based economic evaluation is that it was based on a rigorously conducted Randomised Controlled Trial (RCT). The cost and outcome data measures that were incorporated into the model were collected prospectively during the RCT using forms filled out at the pre-, intra-, and post-operative phase and at the time of discharge from hospital. In addition the economic evaluation benefitted from significant clinical and statistical input throughout its design and development. All assumptions used in the model were agreed with the trial team before the analysis was carried out and without knowledge of how these assumptions would affect the results.

In terms of limitations, it was not possible to account for long-term implications relating to maternal fetal exposure as data relating to this wasn't available from the trial. In addition, information relating to the clinical status and care of the infant was not included in the analysis. A further limitation of the study is that outcomes were expressed in terms of clinical effectiveness rather than in terms of a standard unit of benefit such as the quality-adjusted life-year (QALY). Finally, the use of platelets and other blood products has not been included in the study. However, the results of the sensitivity and threshold analyses

For peer review only - http://bmjopen.bmj.com/site/about/guidelines.xhtml

demonstrated that including these costs would not have impacted on the cost-effectiveness results.

Strengths and weaknesses in relation to other studies

To date, there has only been one, small, RCT looking at the elective use of cell salvage at caesarean section[24] and this study did not include an economic element. A Cochrane review of cell salvage in adult elective surgery assessed the clinical and cost-effectiveness of cell salvage and other autologous transfusion strategies in elective surgery[10]. It suggested that cell salvage may be an 'effective and cost-effective alternative to the allogeneic blood transfusion strategy'. However, no obstetric papers were identified for this review. A recent study by Lim et al [25] found the use of cell salvage for cases at high risk for obstetric hemorrhage to be economically reasonable while routine cell salvage use for all caesarean deliveries was not. This study adopted a societal perspective and used data from published research to populate the model.

Meaning of the study

The results of the economic evaluation suggest that while routine cell salvage is a marginally more effective strategy than standard care in avoiding a donor blood transfusion there is uncertainty in relation to whether it is a less or more costly strategy. Under the conditions reported here, for a high income country (HIC) such as the UK, where donor blood is typically available, cell salvage is unlikely to be considered a cost-effective alternative to the provision of donor blood by the health service. However, in lower and middle income countries (LMIC) where the provision of a safe and secure blood supply may be more challenging the relative cost-effectiveness may be very different. In addition, the lack of long

For peer review only

For peer review only - http://bmjopen.bmj.com/site/about/guidelines.xhtml

term data on the health and quality of life of patients in both arms of the trial means that further research is needed to fully understand the cost implications of both strategies. For example, latent infection such as hepatitis may result in chronic liver disease within 20 or more years of incident of infection [26, 27] which has obvious long term cost implications for the health care provider.

Unanswered questions and future research

The current study has used data from a large, multicentre randomised trial which demonstrated modest evidence that routine use of cell salvage during caesarean section reduced the need for donor blood transfusion. The main cause of uncertainty relates to the cost implications of adopting the routine use of cell salvage. Future studies should explore the long-term health and economic impacts associated with both transfusion strategies. Also, evidence on the preferences of women needs to be considered. For example hospitals may wish to have the option of cell salvage available for Jehovah witness patients where there is no option to use donor blood. In countries where safe donor supply cannot be guaranteed the use of cell salvage might have very different implications which need to be explored.

Finally, the issue of donor blood as a scarce resource needs to be considered. As things currently stand demand for donor blood is increasing while on the other hand enhanced safety measures are limiting the donor pool [28, 29]. The impact of further restrictions on supply could create shortages under current usage patterns, and donor blood substitutes such as cell salvage play a potential role in helping to re-establish a demand-supply balance [30, 31]. Not considered in this study is that fact that transfusion with cell salvage can

For peer review only

For peer review only - http://bmjopen.bmj.com/site/about/guidelines.xhtml

always exist. While there is an expectation that donor blood will always be there when needed, transfusion using donor blood simply cannot be guaranteed. In such a scenario, where the option of donor blood is limited or not available, the routine use of cell salvage would be dominant (less costly and more effective) compared to standard care thus making provision for the availability of the technology likely to be extremely important.

For peer review only

Acknowledgments: The study group would like to thank local midwifery, theatre obstetric, and anaesthetic teams for their support, as well as members of the trial steering and data monitoring committees, patient and public representatives, staff at the Women's Health Research Unit and Pragmatic Clinical Trials Unit at Queen Mary University of London, and all the women who agreed to participate in the SALVO trial.

Contributors: CML undertook the economic analysis, interpreted the results, and drafted the manuscript with input from all authors. TR contributed to the trial design, developed the economic analysis plan, supervised the economic analysis and revised the paper. LJ contributed to the economic analysis plan and revised the paper. PM contributed to the trial design, provided clinical advice and revised the paper. MW contributed to the trial design and revised the paper. RH contributed to the trial design, developed the statistical plan and revised the paper. SA contributed to the trial design and revised the paper. IW contributed to the trial design and revised the paper. LB performed the trial statistical analysis and revised the paper. JG, JD, SC, AV, PA, SR, FGS and MH contributed to the trial design and revised the paper. DL supervised the general management of the trial, supervised data collection and revised the paper. JD supervised the running of the tiral and revised the paper. KK was the chief investigator for the trial, contributed to the trial design, managed the trial and revised the paper.

For peer review only

REFERENCES

1. Centre for Maternal and Child Enquiries (CMAEC), *Saving Mothers' Lives: reviewing maternal deaths to make motherhood safer: 2006–08. The Eighth Report on Confidential Enquiries into Maternal Deaths in the United Kingdom.* BJOG: an International Journal of Obstetrics and Gynaecology, 2011. **118**(Suppl. 1).
2. Bodelon, C., et al., *Factors associated with peripartum hysterectomy.* Obstetrics and gynecology, 2009. **114**(1): p. 115.
3. Intensive Care National Audit & Research Centre (ICNARC), *Female Admissions (Aged 16-50 years) to Adult, General Critical Care Units in England, Wales and Northern Ireland Reported as 'Currently Pregnant' or 'Recently Pregnant'*. 2013.
4. Bateman, B.T., et al., *The epidemiology of postpartum hemorrhage in a large, nationwide sample of deliveries.* Anesthesia & Analgesia, 2010. **110**(5): p. 1368-1373.
5. NHS Blood and Transplant.*Saving and Improving Lives. Strategic Plan 2015-20*.2015 [accessed:September 18 2017] Available from: https://nhsbtdbe.blob.core.windows.net/umbraco-assets-corp/1593/strategic_plan_2015_20.pdf
6. Catling, S., *Intraoperative cell salvage in obstetrics.* Clinical Risk, 2008. **14**(1): p. 14-17.
7. Mehra, T., et al., *Implementation of a patient blood management monitoring and feedback program significantly reduces transfusions and costs.* Transfusion, 2015. **55**(12): p. 2807-2815.
8. Shander, A., J. Isbister, and H. Gombotz, *Patient blood management: the global view.* Transfusion, 2016. **56**(S1).
9. Carless, P.A., et al., *Cell salvage for minimising perioperative allogeneic blood transfusion.* Cochrane Database Syst Rev, 2010. **4**(4).
10. Davies, L., et al., *Cost-effectiveness of cell salvage and alternative methods of minimising perioperative allogeneic blood transfusion: a systematic review and economic model.* Health Technology Assessment, 2006. **10**(44).
11. Khan, K.S., et al., *A randomised controlled trial and economic evaluation of intraoperative cell salvage during caesarean section in women at risk of haemorrhage: the SALVO (cell SALVage in Obstetrics) trial.* Health Technol Assess 2018. **22**(2).
12. National Institute for Health and Care Excellence (NICE), *Costing Statement: Blood Transfusion. Implementing the NICE Guideline on Blood Transfusion (NG24).* 2015.
13. NHS Supply Chain.*NHS Supply Chain Catalogue*.2015 [accessed:August 2016] Available from: https://my.supplychain.nhs.uk/catalogue
14. UK Cell Salvage Action Group, *ICS Technical Fact Sheet: Swab Washing*. 2015.
15. Committee, J.F.*British National Formulary*.2016 [accessed:August 2016] Available from: https://bnf.nice.org.uk/
16. Curtis L, *Unit Costs of Health and Social Care 2015*. 2015, University of Kent: PSSRU.
17. Qureshi, H., et al., *BCSH guideline for the use of anti-D immunoglobulin for the prevention of haemolytic disease of the fetus and newborn.* Transfusion Medicine, 2014. **24**(1): p. 8-20.
18. NHS Blood and Transplant.*Blood and Components Price List 2016/2017*.2016 [accessed:September 18 2017] Available from: http://hospital.blood.co.uk/media/28230/component-price-list-2016-2017.pdf
19. Department of Health.*NHS Reference Costs 2014/2015*.2015 [accessed:August 2016] Available from: https://www.gov.uk/government/uploads/system/uploads/attachment_data/file/477919/2014-15_Reference_costs_publication.pdf
20. NHS National Tariff Payment System.*2016/17 National Prices*.2016 [accessed:August 2016] Available from: www.gov.uk/government/publications/national-tariff-payment-system-2014-to-2015

For peer review only - http://bmjopen.bmj.com/site/about/guidelines.xhtml

21. Tinegate, H., et al., *Guideline on the investigation and management of acute transfusion reactions Prepared by the BCSH Blood Transfusion Task Force.* British journal of haematology, 2012. **159**(2): p. 143-153.
22. National Institute for Health and Care Excellence (NICE), *Guide to the methods of technology appraisal*. 2013, Springer.
23. Gupta, S.K., *Intention-to-treat concept: a review.* Perspectives in clinical research, 2011. **2**(3): p. 109.
24. Rainaldi, M., et al., *Blood salvage during caesarean section.* British journal of anaesthesia, 1998. **80**(2): p. 195-198.
25. Lim, G., et al., *Cost-effectiveness analysis of intraoperative cell salvage for obstetric hemorrhage.* Anesthesiology: The Journal of the American Society of Anesthesiologists, 2018. **128**(2): p. 328-337.
26. Seeff, L.B., et al., *Long-term mortality after transfusion-associated non-A, non-B hepatitis.* New England Journal of Medicine, 1992. **327**(27): p. 1906-1911.
27. Shalmani, H.M., M. Ranjbar, and A.H.M. Alizadeh, *Recommendations for prevention and control of hepatitis C virus (HCV) infection and HCV-related chronic disease.* J Liver, 2013. **3**(147): p. 2167-0889.1000147.
28. Busch, M.P., S.H. Kleinman, and G.J. Nemo, *Current and emerging infectious risks of blood transfusions.* Jama, 2003. **289**(8): p. 959-962.
29. Williamson, L.M. and D.V. Devine, *Challenges in the management of the blood supply.* The Lancet, 2013. **381**(9880): p. 1866-1875.
30. Spahn, D.R. and L.T. Goodnough, *Alternatives to blood transfusion.* The Lancet, 2013. **381**(9880): p. 1855-1865.
31. Dixon, S., et al., *Economic analysis of the implementation of autologous transfusion technologies throughout England.* International journal of technology assessment in health care, 2005. **21**(2): p. 234-239.

For peer review only

For peer review only - http://bmjopen.bmj.com/site/about/guidelines.xhtml

TABLES

Table 1 Probabilities used in the model
Table 2 Intraoperative resource use and costs
Table 3 Postoperative resource use and costs
Table 4 Results
Table 5 Deterministic sensitivity analysis

FIGURES

Figure 1 Decision tree structure
Figure 2 Cost-effectiveness acceptability curves for donor blood transfusion avoided

For peer review only

For peer review only - http://bmjopen.bmj.com/site/about/guidelines.xhtml

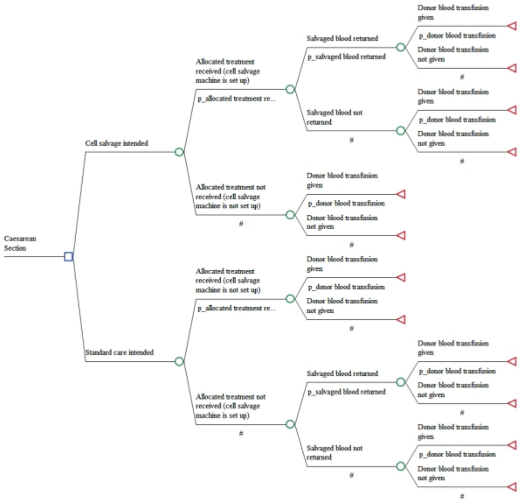

Decision tree structure

167x86mm (300 x 300 DPI)

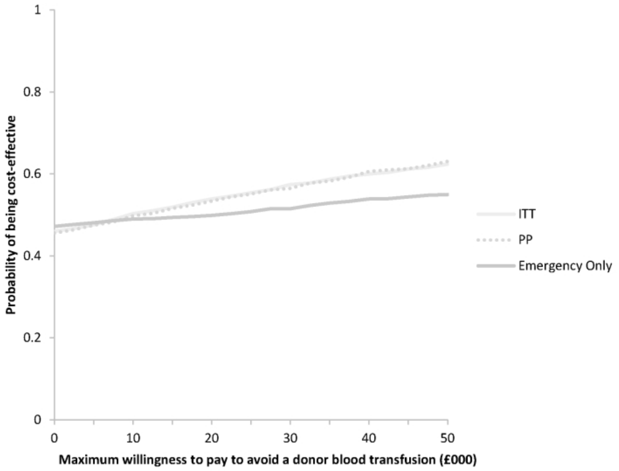

Cost-effectiveness acceptability curves for donor blood transfusion avoided

210x297mm (300 x 300 DPI)

The CHEERS Checklist is part of the CHEERS Statement. The CHEERS Statement has been endorsed and co-published by the following journals:

BJOG: An International Journal of Obstetrics and Gynaecology
BMC Medicine 2013; 11:80
BMJ 2013;346:f1049
Clinical Therapeutics 27 March 2013 (Article in Press DOI: 10.1016/j.clinthera.2013.03.003)
Cost Effectiveness and Resource Allocation 2013 11:6.
The European Journal of Health Economics 2013 Mar 26. [Epub ahead of print]
International Journal of Technology Assessment in Health Care
Journal of Medical Economics 2013 Mar 25. [Epub ahead of print]
Pharmacoeconomics 2013 Mar 26. [Epub ahead of print]
Value in Health 2013 March - April;16(2):e1-e5

CHEERS Checklist
Items to include when reporting economic evaluations of health interventions

Section/item	Item No	Recommendation	Reported on page No/ line No
**Title and abstract**			
Title	1	Identify the study as an economic evaluation or use more specific terms such as "cost-effectiveness analysis", and describe the interventions compared.	1/1
Abstract	2	Provide a structured summary of objectives, perspective, setting, methods (including study design and inputs), results (including base case and uncertainty analyses), and conclusions.	3/32
**Introduction**			
Background and objectives	3	Provide an explicit statement of the broader context for the study. Present the study question and its relevance for health policy or practice decisions.	6/92
**Methods**			
Target population and subgroups	4	Describe characteristics of the base case population and subgroups analysed, including why they were chosen.	7/118
Setting and location	5	State relevant aspects of the system(s) in which the decision(s) need(s) to be made.	7/118
Study perspective	6	Describe the perspective of the study and relate this to the costs being evaluated.	7/127
Comparators	7	Describe the interventions or strategies being compared and state why they were chosen.	7/121
Time horizon	8	State the time horizon(s) over which costs and consequences are being evaluated and say why appropriate.	7/130
Discount rate	9	Report the choice of discount rate(s) used for costs and	n/a

		outcomes and say why appropriate.	[illegible]
Choice of health outcomes	10	Describe what outcomes were used as the measure(s) of benefit in the evaluation and their relevance for the type of analysis performed.	7 / 128
Measurement of effectiveness	11a	*Single study-based estimates:* Describe fully the design features of the single effectiveness study and why the single study was a sufficient source of clinical effectiveness data.	n/a
	11b	*Synthesis-based estimates:* Describe fully the methods used for identification of included studies and synthesis of clinical effectiveness data.	n/a
Measurement and valuation of preference based outcomes	12	If applicable, describe the population and methods used to elicit preferences for outcomes.	n/a
Estimating resources and costs	13a	*Single study-based economic evaluation:* Describe approaches used to estimate resource use associated with the alternative interventions. Describe primary or secondary research methods for valuing each resource item in terms of its unit cost. Describe any adjustments made to approximate to opportunity costs.	n/a
	13b	*Model-based economic evaluation:* Describe approaches and data sources used to estimate resource use associated with model health states. Describe primary or secondary research methods for valuing each resource item in terms of its unit cost. Describe any adjustments made to approximate to opportunity costs.	7/133
Currency, price date, and conversion	14	Report the dates of the estimated resource quantities and unit costs. Describe methods for adjusting estimated unit costs to the year of reported costs if necessary. Describe methods for converting costs into a common currency base and the exchange rate.	8 / 143
Choice of model	15	Describe and give reasons for the specific type of decision-analytical model used. Providing a figure to show model structure is strongly recommended.	7/133
Assumptions	16	Describe all structural or other assumptions underpinning the decision-analytical model.	7/133
Analytical methods	17	Describe all analytical methods supporting the evaluation. This could include methods for dealing with skewed, missing, or censored data; extrapolation methods; methods for pooling data; approaches to validate or make adjustments (such as half cycle corrections) to a model; and methods for handling population heterogeneity and uncertainty.	9 / 164
**Results**			
Study parameters	18	Report the values, ranges, references, and, if used, probability distributions for all parameters. Report reasons or sources for distributions used to represent uncertainty where appropriate. Providing a table to show the input values is strongly recommended.	7/138

Incremental costs and outcomes	19	For each intervention, report mean values for the main categories of estimated costs and outcomes of interest, as well as mean differences between the comparator groups. If applicable, report incremental cost-effectiveness ratios.	10/202
Characterising uncertainty	20a	*Single study-based economic evaluation:* Describe the effects of sampling uncertainty for the estimated incremental cost and incremental effectiveness parameters, together with the impact of methodological assumptions (such as discount rate, study perspective).	n/a
	20b	*Model-based economic evaluation:* Describe the effects on the results of uncertainty for all input parameters, and uncertainty related to the structure of the model and assumptions.	11/224
Characterising heterogeneity	21	If applicable, report differences in costs, outcomes, or cost-effectiveness that can be explained by variations between subgroups of patients with different baseline characteristics or other observed variability in effects that are not reducible by more information.	n/a
**Discussion**			
Study findings, limitations, generalisability, and current knowledge	22	Summarise key study findings and describe how they support the conclusions reached. Discuss limitations and the generalisability of the findings and how the findings fit with current knowledge.	13/264
**Other**			
Source of funding	23	Describe how the study was funded and the role of the funder in the identification, design, conduct, and reporting of the analysis. Describe other non-monetary sources of support.	4/76
Conflicts of interest	24	Describe any potential for conflict of interest of study contributors in accordance with journal policy. In the absence of a journal policy, we recommend authors comply with International Committee of Medical Journal Editors recommendations.	5/83

For consistency, the CHEERS Statement checklist format is based on the format of the CONSORT statement checklist

The CHEERS Statement may be accessed by the publication links above.

The **ISPOR CHEERS Task Force Report** provides examples and further discussion of the 24-item CHEERS Checklist and the CHEERS Statement. It may be accessed via the *Value in Health* link or via the ISPOR Health Economic Evaluation Publication Guidelines – CHEERS: Good Reporting Practices webpage: http://www.ispor.org/TaskForces/EconomicPubGuidelines.asp

The citation for the CHEERS Task Force Report is:
Husereau D, Drummond M, Petrou S, et al. Consolidated health economic evaluation reporting standards (CHEERS)—Explanation and elaboration: A report of the ISPOR health economic evaluations publication guidelines good reporting practices task force. Value Health 2013;16:231-50.

BMJ Open

Cost-effectiveness of cell salvage and donor blood transfusion during caesarean section: results from a randomised controlled trial

Journal:	*BMJ Open*
Manuscript ID	bmjopen-2018-022352.R2
Article Type:	Research
Date Submitted by the Author:	20-Nov-2018
Complete List of Authors:	McLoughlin, Carol; University of Birmingham, Health Economics Unit<br>Roberts, Tracy; University of Birmingham, Health Economics Unit<br>Jackson, Louise; University of Birmingham, Health Economics Unit<br>Moore, Philip; Birmingham Women's Hospital, Selwyn Crawford Department of Anaesthetics<br>Wilson, Matthew; University of Sheffield, School of Health and Related Research (ScHARR)<br>Hooper, Richard; Queen Mary University of London, Centre for Primary Care & Public Health<br>Allard, Shubha; NHS Blood and Transplant and Barts Health NHS Trust<br>Wrench, Ian; Sheffield Teaching Hospitals NHS Foundation Trust Department of Anaesthesia<br>Beresford, Lee; Queen Mary University of London, Centre for Primary Care & Public Health<br>Geoghegan, James; Birmingham Women's Hospital, Selwyn Crawford Department of Anaesthetics<br>Daniels, Jane; University of Nottingham, Nottingham Clinical Trials Unit<br>Catling, Sue ; Singleton Hospital, Department of Anaesthetics<br>Clark, Vicki; Royal Infirmary of Edinburgh, Simpson Centre for Reproductive Health<br>Ayuk, Paul; Royal Victoria Infirmary, Women's Services<br>Robson, Stephen; NIHR CRN North East and North Cumbria<br>Gao, Fang; University of Birmingham, Institute of Inflammation and Ageing, College of Medical and Dental Sciences; Heart of England NHS Foundation Trust, Academic Department of Anaesthesia, Critical Care, Pain and Resuscitation<br>Hogg, Matthew; Royal London Hospital, Barts Health NHS Trust<br>Lanz, Doris; Queen Mary University of London, Centre for Psychiatry<br>Dodds, Julie; Barts and The London School of Medicine and Dentistry, Women's Health Research Unit<br>Khan, Khalid; Queen Mary, University of London, Centre for Primary Care and Public Health
**Primary Subject Heading**:	Health economics
Secondary Subject Heading:	Obstetrics and gynaecology, Health economics, Haematology (incl blood transfusion)
Keywords:	HEALTH ECONOMICS, Clinical trials < THERAPEUTICS, Maternal medicine

	< OBSTETRICS, Blood bank & transfusion medicine < HAEMATOLOGY, Health economics < HEALTH SERVICES ADMINISTRATION & MANAGEMENT

SCHOLARONE™
Manuscripts

Cost-effectiveness of cell salvage and donor blood transfusion during caesarean section: results from a randomised controlled trial

Carol McLoughlin[1], Tracy E Roberts[1*], Louise J Jackson[1], Philip Moore,[2] Matthew Wilson,[3] Richard Hooper,[4] Shubha Allard,[5] Ian Wrench,[6] Lee Beresford,[4] James Geoghegan,[2] Jane Daniels,[7] Sue Catling,[8] Vicki A Clark,[9] Paul Ayuk,[10] Stephen Robson,[11] Fang Gao,[12] Matthew Hogg,[13] Doris Lanz,[14] Julie Dodds,[14] Khalid Khan[14] on behalf of the SALVO study group

[1] Health Economics Unit, University of Birmingham, B15 2TT UK

[2] Birmingham Women's Hospital, Birmingham, B15 2TH UK

[3] School of Health and Related Research (ScHARR), University of Sheffield, S1 4DA UK

[4] Pragmatic Clinical Trials Unit, Centre for Primary Care and Public Health, Queen Mary University of London, E1 2AB UK

[5] NHS Blood and Transplant, London, NW9 5BG UK

[6] Sheffield Teaching Hospitals NHS Foundation Trust, Sheffield, S10 2JF UK

[7] Nottingham Clinical Trials Unit, University of Nottingham, NG7 2UH UK

[8] Singleton Hospital, Swansea, SA2 8QA UK

[9] Simpson Centre for Reproductive Health, Royal Infirmary, Edinburgh, EH16 4SA UK

[10] Royal Victoria Infirmary, Newcastle-upon-Tyne, NE1 4LP UK

[11] Institute of Cellular Medicine, Newcastle University, NE2 4HH UK

[12] Perioperative, Critical Care and Trauma Trials Group, University of Birmingham, B15 2WB UK

[13] Royal London Hospital, Barts Health NHS Trust, London, E1 1BB UK

For peer review only - http://bmjopen.bmj.com/site/about/guidelines.xhtml

[14] Women's Health Research Unit, Barts and the London School of Medicine and Dentistry,
Queen Mary University of London, E1 2AB UK

*Corresponding Author
Email: t.e.roberts@bham.ac.uk

For peer review only

For peer review only - http://bmjopen.bmj.com/site/about/guidelines.xhtml

ABSTRACT

Objectives: To evaluate the cost-effectiveness of routine use of cell salvage during caesarean section in mothers at risk of haemorrhage compared with current standard of care.

Design: Model-based cost-effectiveness evaluation alongside a multicentre randomised controlled trial (RCT). Three main analyses were carried out on the trial data: (1) based on the intention to treat principle; (2) based on the per protocol principle; (3) only participants who underwent an emergency caesarean section.

Setting: 26 obstetric units in the UK.

Participants: 3028 women at risk of haemorrhage recruited between June 2013 and April 2016.

Interventions: Cell salvage (intervention) versus routine care without salvage (control).

Primary outcome measures: Cost-effectiveness based on incremental cost per donor blood transfusion avoided.

Results: In the intention to treat analysis the mean difference in total costs between cell salvage and standard care was £83. The estimated incremental cost-effectiveness ratio (ICER) was £8,110 per donor blood transfusion avoided. For the per protocol analysis the mean difference in total costs was £92 and the ICER was £8,252. In the emergency caesarean section analysis the mean difference in total costs was £55 and the ICER was £13,713 per donor blood transfusion avoided. This ICER is driven by the increased probability that these patients would require a higher level of post-operative care and additional surgeries. The results of these analyses were shown to be robust for the majority of deterministic sensitivity analyses.

For peer review only

Conclusions: The results of the economic evaluation suggest that while routine cell salvage is a marginally more effective strategy than standard care in avoiding a donor blood transfusion there is uncertainty in relation to whether it is a less or more costly strategy. The lack of long term data on the health and quality of life of patients in both arms of the trial means that further research is needed to fully understand the cost implications of both strategies.

Trial registration number: Current Controlled Trials ISRCTN66118656

STRENGTHS AND LIMITATIONS OF THIS STUDY

- Study strengths of this model-based economic evaluation include that it was based on a rigorously conducted RCT and it benefitted from significant clinical and statistical input throughout its design and development.
- The analyses were conducted from a healthcare perspective, and the cost and outcome data measures incorporated into the model were collected prospectively during the RCT using forms filled out during the pre-intra- and postoperative phases and at the time of discharge from hospital.
- A limitation of the study is that not all potential outcomes have been included because of the limited time scale in the model and the lack of long-term data.
- A further limitation of the evaluation is that outcomes were expressed in terms of clinical effectiveness rather than in terms of a standard unit of benefit such as the QALY (quality-adjusted life year).

Funding: Funding for this study was provided by the Health Technology Assessment Programme of the National Institute for Health Research. Project number 10/57/32.

For peer review only - http://bmjopen.bmj.com/site/about/guidelines.xhtml

Disclaimer: The views and opinions expressed herein are those of the authors and do not necessarily reflect those of the Health Technology Assessment programme, NIHR, NHS or the Department of Health.

Competing interests: None declared.

Data sharing statement: No additional data available.

Word Count: 3,442 words (not including title page, abstract, references, tables, figures)

Reporting statement: Consolidated Health Economic Evaluation Reporting Standards (CHEERS) statement provided.

For peer review only - http://bmjopen.bmj.com/site/about/guidelines.xhtml

INTRODUCTION

Excessive blood loss (haemorrhage) in childbirth is a life threatening condition which is an important cause of maternal death[1], emergency hysterectomy[2], and maternal critical care admission[3] among women undergoing a caesarean section[4]. The treatment for major haemorrhage includes donor blood transfusion. However, red cell concentrates used in donor transfusion are a scarce, nationally pooled resource in demand simultaneously by many clinical services[5]. Furthermore such transfusions carry risks for recipients as a result of incompatibility or infection[6]. Consequently there is a recognised need to make caesarean safer while at the same time promoting the use of transfusion alternatives harnessing the patient’s own reserves where feasible[7, 8].

Intraoperative cell salvage (IOCS), which collects, processes, and returns the woman’s own blood lost during surgery, is increasingly being deployed during caesareans. It has been shown to reduce the need for donor blood transfusions in a wide spectrum of surgical disciplines[9, 10].

This study presents the results of a model based economic evaluation which compared the relative cost and cost-effectiveness of routine cell salvage with standard transfusion with donor blood in caesarean section. This study is part a wider NIHR-HTA RCT exploring the effectiveness and cost-effectiveness of the use of cell salvage compared to donor blood transfusion during caesarean section[11]. Primary data on costs and resource use were collected prospectively alongside the trial and the principal outcome for the economic evaluation was the cost per donor blood transfusion avoided.

For peer review only - http://bmjopen.bmj.com/site/about/guidelines.xhtml

METHODS

Full details of the randomised controlled trial (RCT) are reported elsewhere[11]. Briefly, the study was performed in 26 obstetric units. The sample comprised women who were admitted to a participating labour ward and who fulfilled all of the following inclusion criteria: aged $\geq$ 16 years; able to provide informed consent; undergoing delivery by caesarean section with an identifiable increased risk of haemorrhage, defined as all emergency caesarean sections, where maternal or fetal compromise is suspected, and elective caesarean section for all indications other than maternal request or breech presentation. Participating women were randomised to either caesarean section with cell salvage (intervention group), or to caesarean section without cell salvage (control group) for which transfusion of donor blood was carried out according to local guidelines. The primary outcome for the RCT was the proportion of women receiving donor blood transfusion due to haemorrhage. For the economic analysis a decision analytic model was deemed the most suitable method of presenting the alternative pathways and collating the data for analysis. The economic evaluation took the form of a cost-effectiveness analysis from the perspective of the health care provider based on the principal clinical outcome of the trial i.e. the proportion of women receiving donor blood transfusion due to haemorrhage. Given the objectives of the trial and the duration of follow up, outcomes beyond the trial endpoint were not considered plausible or relevant in the model based analysis.

Model structure

A decision tree model was developed in TreeAge Pro 2016 (TreeAge Software, Inc., Williamstown, MA, USA) to represent the alternative strategies. The pathways of the model (see Figure 1) represent, as far as possible, the clinical procedures carried out in the study.

For peer review only - http://bmjopen.bmj.com/site/about/guidelines.xhtml

Clinical data used in the model

The model combines the probability of a woman following a particular path and the associated costs. Probabilities, detailed in Table 1, were obtained from the trial and attached to each pathway.

Resource use and costs

The resource use for both arms of the trial was estimated by evaluating the individual components of these procedures (bottom-up costing). Unit cost data was then attached to the resource use. Data were collected on all major NHS resource use for each patient using the trial case report forms.

For the analysis, intraoperative and postoperative resource use data were obtained from the SALVO trial. The main resource use monitored included: equipment and disposables required for the cell salvage procedure; additional staff called into theatre solely for the purposes of cell salvage; drugs used in the caesarean section procedure; length and type of hospital inpatient stay including any additional treatment required attributed to the caesarean procedure; the use of donor blood transfusion and salvaged blood transfusion to deal with haemorrhage and its consequences.

Intra-operative costs were estimated for each item to arrive at a mean cost per caesarean procedure for each treatment pathway in the model. To estimate the cost of a caesarean section procedure some costs were calculated at the patient level e.g. swab washing and

For peer review only - http://bmjopen.bmj.com/site/about/guidelines.xhtml

For peer review only

some at the procedural level e.g. drugs used in the caesarean section procedure (see Table 2). Postoperative costs were estimated for each item based on their occurrence in each brand of the model to arrive at a mean cost per patient for each branch (see Table 3).

ANALYSIS

Given the objectives of the trial and the duration of follow up, a within trial economic analysis was carried out. The analysis took the perspective of the NHS and PSS following current recommendations from NICE[12]. The main economic analysis was a cost-effectiveness analysis (CEA) with results expressed as cost per donor transfusion avoided.

We carried out three main analyses on the trial data. The first analysis was based on the intention-to-treat (ITT) principle (Analysis ITT). In this method, patients are compared within the treatment groups to which they were originally randomised irrespective of the treatment received[13]. This method of analysis allows the estimates to follow real-life scenarios in which patients may not always receive the planned treatment. ITT is recommended to ensure that the benefits of a given intervention are not exaggerated [13]. The second analysis was based on the treatment actually received by patients irrespective of randomisation (Analysis 'per protocol' (PP))). Within the SALVO trial, equal numbers of patients were randomised to either cell salvage or standard care. However, because some clinicians managing women in the control arm had access to a cell salvage machine, it was possible that women in the control arm could receive cell salvage in place of a donor blood transfusion. A PP analysis was carried out to look at the effect of treatment received on the outcome estimates. Therefore, in Analysis PP all patients who received cell salvage were compared with those who received standard care, irrespective of the treatment to which

For peer review only - http://bmjopen.bmj.com/site/about/guidelines.xhtml

they were randomised. The third analysis considered only patients who underwent an emergency caesarean section (Analysis ECS). This analysis was considered necessary as the SALVO trial found that numerically, there was a greater reduction in the rate of transfusion within the emergency patient group compared to the elective patient group. This analysis followed the same methodology as the ITT and PP analyses. Deterministic and probabilistic sensitivity analyses (PSAs) were carried out for each analysis to explore the effects of the inherent uncertainty in parameter estimates on model results.

Patient and public involvement

The UK National Childbirth Trust collaborated in the project by providing patient and public input through involvement in trial design and protocol development. Prior to the SALVO trial, a survey was conducted among women who received cell salvage, showing that they perceived the interventions as reassuring, safe, ad preferable to donor blood transfusion. A patient representative was a member of the trial steering committee to provide oversight and advice regarding recruitment, dissemination, and general trial management. We are planning to disseminate findings to participants in the form of a newsletter following primary publication of these results.

RESULTS

The results of the ITT analysis (Analysis ITT) suggest that routine cell salvage is more costly than standard care with the mean difference in total costs per patient estimated at £83. The Incremental Cost-Effectiveness Ratio (ICER) representing the additional cost of routine cell salvage during caesarean section in women at risk of haemorrhage compared to standard care for these women was estimated to be approximately £8,110 per donor blood

For peer review only

transfusion avoided. The overall result, based on the PP analysis (Analysis PP), was an ICER of £8,252 per transfusion avoided for cell salvage compared to standard care. The emergency caesarean analysis (Analysis ECS) resulted in an ICER of £13,713 per transfusion avoided. See Table 4.

The results of the corresponding PSAs are presented using cost-effectiveness acceptability curves (CEACs) to graphically represent uncertainty in the appropriate threshold cost-effectiveness value (Figure 2). The CEAC for Analysis ITT shows that the probability that routine cell salvage is cost-effective increases as the willingness to pay for a donor blood transfusion avoided increases. If the maximum willingness to pay for a donor blood transfusion avoided was £50,000, for example, the probability of routine cell salvage being cost-effective would be 62%. For Analysis PP the probability that routine cell salvage was cost-effective at a willingness to pay of £50,000, would be 63%. The CEAC for Analysis ECS shows that the probability that cell salvage is cost-effective remains between 47% and 55% as the willingness to pay for a donor blood transfusion avoided increases.

Deterministic sensitivity analysis

A number of deterministic sensitivity analyses were conducted in each analysis (see Table 5):

(i) The main analyses used costs for consumables based on a particular model of the cell saver machine. To assess the difference that variation in these estimates would make, the unit costs were replaced with unit costs obtained from the NICE costing statement for blood transfusion[14]. This had a marginal impact on the ICER in each analysis. The impact of the inclusion of acquisition costs for a cell salvage machine

For peer review only - http://bmjopen.bmj.com/site/about/guidelines.xhtml

For peer review only

was also explored and found this had only a minimal impact on the ICER in each analysis. In the trial, 202 cases used a cell salvage machine that required consumables for collection only, even where the blood was not processed. The impact of including costs for the consumables used by this machine, where the machine is only set up for processing in patients having blood returned and where swab washing is not conducted, resulted in an ICER of £1,022 in Analysis ITT, £1,184 in Analysis PP. In Analysis ECS there was a dominant result in that cell salvage was considered less costly and more effective compared to standard care.

(ii) The base case analyses used the mean length of time additional staff were present in theatre in each arm solely for the purposes of cell salvage. In the sensitivity analysis the cost of additional staff was removed. This reduced the ICER to £7,065 in Analysis ITT, £7,210 in Analysis PP, and £10,932 in Analysis ECS.

(iii) To facilitate robust evaluation in cost-effectiveness analyses relating to donor blood, a comprehensive estimate for the cost of a unit of donor blood is required. The NHS Blood and Transplant Authority (NHSBTA) have valued the cost of RBC to be £120 per unit based on direct costs to the healthcare services[15]. However, there is significant uncertainty surrounding this figure. We conducted a study (submitted for publication) parallel to the SALVO trial that aimed to dissect the current price of blood. We explored what elements are contributing to the current cost of blood and what elements are missing. Our study concluded that the current costing approach of assuming there will always be an adequate supply of donor blood must be replaced with including provisions for the continued shrinking of the donor pool and the impact that future shocks to the blood supply system could have. This sensitivity analysis assessed the difference that variation in the estimated cost of blood made

For peer review only - http://bmjopen.bmj.com/site/about/guidelines.xhtml

to the overall cost-effectiveness of routine cell salvage. Raising the cost of a three unit transfusion of RBC to a hypothetical cost of £1,500 reduced the ICER by £974 in both the ITT and PP analysis and it reduced the ICER by over £1,000 in the emergency caesarean analysis. Threshold analysis showed that for routine cell salvage to be considered cost-effective, the cost of an RBC transfusion would have to increase to £8,637 in Analysis ITT, £8,778 in Analysis PP, and £13,186 in Analysis ECS.

DISCUSSION

Principal findings

The results of this economic evaluation suggest that routine cell salvage is more costly than standard care with the average cost per patient estimated at £1,327. The ICER for this strategy compared with standard care is approximately £8,110 per donor blood transfusion avoided. The Probabilistic Sensitivity Analysis suggests that at an arbitrary willingness to pay threshold of £50,000, the probability of routine cell salvage being cost-effective is 62%.

The results of this analysis were shown to be robust for the majority of deterministic sensitivity analyses with one exception; using a cell salvage machine that required different consumables to those included in the main base case analyses,where the machine is only set up for processing in patients having blood returned and where swab washing is not conducted, resulted in a significant effect on the ICER, reducing it to £1,022 per donor blood transfusion avoided.

A per protocol analysis produced an ICER of £8,252 per transfusion avoided, but this result should be considered with caution as the population in this analysis is a subset of the ITT

For peer review only - http://bmjopen.bmj.com/site/about/guidelines.xhtml

population who completed the study without any major protocol violations[13]. In clinical practice uptake of cell salvage is unlikely to be “per-protocol”. In a third analysis, looking at emergency caesareans only, cell salvage appears to be more effective than standard care for avoiding a donor blood transfusion but the resulting ICER of £13,713 is driven by the increased probability that these patients will require a higher level of post-operative care and additional surgeries.

Strengths and limitations of the study

The strength of this model based economic evaluation is that it was based on a rigorously conducted RCT. The cost and outcome data measures that were incorporated into the model were collected prospectively during the RCT using forms filled out at the pre-, intra-, and post-operative phase and at the time of discharge from hospital. In addition the economic evaluation benefitted from significant clinical and statistical input throughout its design and development. All assumptions used in the model were agreed with the trial team before the analysis was carried out and without knowledge of how these assumptions would affect the results.

In terms of limitations, it was not possible to account for long-term implications relating to maternal fetal exposure as data relating to this wasn’t available from the trial. In addition, information relating to the clinical status and care of the infant was not included in the analysis. A further limitation of the study is that outcomes were expressed in terms of clinical effectiveness rather than in terms of a standard unit of benefit such as the quality-adjusted life-year (QALY). Finally, the use of platelets and other blood products has not been included in the study. However, the results of the sensitivity and threshold analyses

For peer review only - http://bmjopen.bmj.com/site/about/guidelines.xhtml

demonstrated that including these costs would not have impacted on the cost-effectiveness results.

Strengths and weaknesses in relation to other studies

To date, there has only been one, small, RCT looking at the elective use of cell salvage at caesarean section[16] and this study did not include an economic element. A Cochrane review of cell salvage in adult elective surgery assessed the clinical and cost-effectiveness of cell salvage and other autologous transfusion strategies in elective surgery[10]. It suggested that cell salvage may be an 'effective and cost-effective alternative to the allogeneic blood transfusion strategy'. However, no obstetric papers were identified for this review. A recent study by Lim et al [17] found the use of cell salvage for cases at high risk for obstetric hemorrhage to be economically reasonable while routine cell salvage use for all caesarean deliveries was not. In contrast to the current study, Lim et al. adopted a societal perspective and used data from published research to populate the model [17].

Meaning of the study

The results of the economic evaluation suggest that while routine cell salvage is a marginally more effective strategy than standard care in avoiding a donor blood transfusion there is uncertainty in relation to whether it is a less or more costly strategy. Under the conditions reported here, for a high income country (HIC) such as the UK, where donor blood is typically available, cell salvage is unlikely to be considered a cost-effective alternative to the provision of donor blood by the health service. However, in lower and middle income countries (LMIC) where the provision of a safe and secure blood supply may be more challenging the relative cost-effectiveness may be very different. In addition, the lack of long

For peer review only - http://bmjopen.bmj.com/site/about/guidelines.xhtml

term data on the health and quality of life of patients in both arms of the trial means that further research is needed to fully understand the cost implications of both strategies. For example, latent infection such as hepatitis may result in chronic liver disease within 20 or more years of incident of infection [18, 19] which has obvious long term cost implications for the health care provider.

Unanswered questions and future research

The current study has used data from a large, multicentre randomised trial which demonstrated modest evidence that routine use of cell salvage during caesarean section reduced the need for donor blood transfusion. The main cause of uncertainty relates to the long term cost implications of adopting the routine use of cell salvage. Future studies should explore the long-term health and economic and quality of life impacts associated with both transfusion strategies. Also, evidence on the preferences of women needs to be considered. For example hospitals may wish to have the option of cell salvage available for Jehovah witness patients where there is no option to use donor blood. In countries where safe donor supply cannot be guaranteed the use of cell salvage might have very different implications which need to be explored.

Finally, the issue of donor blood as a scarce resource needs to be considered. As things currently stand demand for donor blood is increasing while on the other hand enhanced safety measures are limiting the donor pool [20, 21]. The impact of further restrictions on supply could create shortages under current usage patterns, and donor blood substitutes such as cell salvage play a potential role in helping to re-establish a demand-supply balance [22, 23]. Not considered in this study is that fact that transfusion with cell salvage can

For peer review only

always exist. While there is an expectation that donor blood will always be there when needed, transfusion using donor blood simply cannot be guaranteed. In such a scenario, where the option of donor blood is limited or not available, the routine use of cell salvage would be dominant (less costly and more effective) compared to standard care thus making provision for the availability of the technology likely to be extremely important.

For peer review only

For peer review only - http://bmjopen.bmj.com/site/about/guidelines.xhtml

Acknowledgments: The study group would like to thank local midwifery, theatre obstetric, and anaesthetic teams for their support, as well as members of the trial steering and data monitoring committees, patient and public representatives, staff at the Women's Health Research Unit and Pragmatic Clinical Trials Unit at Queen Mary University of London, and all the women who agreed to participate in the SALVO trial.

Contributors: CML undertook the economic analysis, interpreted the results, and drafted the manuscript with input from all authors. TR contributed to the trial design, developed the economic analysis plan, supervised the economic analysis and revised the paper. LJ contributed to the economic analysis plan and revised the paper. PM contributed to the trial design, provided clinical advice and revised the paper. MW contributed to the trial design and revised the paper. RH contributed to the trial design, developed the statistical plan and revised the paper. SA contributed to the trial design and revised the paper. IW contributed to the trial design and revised the paper. LB performed the trial statistical analysis and revised the paper. JG, JD, SC, AV, PA, SR, FGS and MH contributed to the trial design and revised the paper. DL supervised the general management of the trial, supervised data collection and revised the paper. JD supervised the running of the tiral and revised the paper. KK was the chief investigator for the trial, contributed to the trial design, managed the trial and revised the paper.

For peer review only - http://bmjopen.bmj.com/site/about/guidelines.xhtml

REFERENCES

1. Centre for Maternal and Child Enquiries (CMAEC), *Saving Mothers' Lives: reviewing maternal deaths to make motherhood safer: 2006–08. The Eighth Report on Confidential Enquiries into Maternal Deaths in the United Kingdom.* BJOG: an International Journal of Obstetrics and Gynaecology, 2011. **118**(Suppl. 1).
2. Bodelon, C., et al., *Factors associated with peripartum hysterectomy.* Obstetrics and gynecology, 2009. **114**(1): p. 115.
3. Intensive Care National Audit & Research Centre (ICNARC), *Female Admissions (Aged 16-50 years) to Adult, General Critical Care Units in England, Wales and Northern Ireland Reported as 'Currently Pregnant' or 'Recently Pregnant'*. 2013.
4. Bateman, B.T., et al., *The epidemiology of postpartum hemorrhage in a large, nationwide sample of deliveries.* Anesthesia & Analgesia, 2010. **110**(5): p. 1368-1373.
5. NHS Blood and Transplant.*Saving and Improving Lives. Strategic Plan 2015-20.*2015 [accessed:September 18 2017] Available from: https://nhsbtdbe.blob.core.windows.net/umbraco-assets-corp/1593/strategic_plan_2015_20.pdf
6. Catling, S., *Intraoperative cell salvage in obstetrics.* Clinical Risk, 2008. **14**(1): p. 14-17.
7. Mehra, T., et al., *Implementation of a patient blood management monitoring and feedback program significantly reduces transfusions and costs.* Transfusion, 2015. **55**(12): p. 2807-2815.
8. Shander, A., J. Isbister, and H. Gombotz, *Patient blood management: the global view.* Transfusion, 2016. **56**(S1).
9. Carless, P.A., et al., *Cell salvage for minimising perioperative allogeneic blood transfusion.* Cochrane Database Syst Rev, 2010. **4**(4).
10. Davies, L., et al., *Cost-effectiveness of cell salvage and alternative methods of minimising perioperative allogeneic blood transfusion: a systematic review and economic model.* Health Technology Assessment, 2006. **10**(44).
11. Khan, K.S., et al., *A randomised controlled trial and economic evaluation of intraoperative cell salvage during caesarean section in women at risk of haemorrhage: the SALVO (cell SALVage in Obstetrics) trial.* Health Technol Assess 2018. **22**(2).
12. National Institute for Health and Care Excellence (NICE), *Guide to the methods of technology appraisal*. 2013, Springer.
13. Gupta, S.K., *Intention-to-treat concept: a review.* Perspectives in clinical research, 2011. **2**(3): p. 109.
14. National Institute for Health and Care Excellence (NICE), *Costing Statement: Blood Transfusion. Implementing the NICE Guideline on Blood Transfusion (NG24).* 2015.
15. NHS Blood and Transplant.*Blood and Components Price List 2016/2017.*2016 [accessed:September 18 2017] Available from: http://hospital.blood.co.uk/media/28230/component-price-list-2016-2017.pdf
16. Rainaldi, M., et al., *Blood salvage during caesarean section.* British journal of anaesthesia, 1998. **80**(2): p. 195-198.
17. Lim, G., et al., *Cost-effectiveness analysis of intraoperative cell salvage for obstetric hemorrhage.* Anesthesiology: The Journal of the American Society of Anesthesiologists, 2018. **128**(2): p. 328-337.
18. Seeff, L.B., et al., *Long-term mortality after transfusion-associated non-A, non-B hepatitis.* New England Journal of Medicine, 1992. **327**(27): p. 1906-1911.
19. Shalmani, H.M., M. Ranjbar, and A.H.M. Alizadeh, *Recommendations for prevention and control of hepatitis C virus (HCV) infection and HCV-related chronic disease.* J Liver, 2013. **3**(147): p. 2167-0889.1000147.

For peer review only

For peer review only - http://bmjopen.bmj.com/site/about/guidelines.xhtml

20. Busch, M.P., S.H. Kleinman, and G.J. Nemo, *Current and emerging infectious risks of blood transfusions.* Jama, 2003. **289**(8): p. 959-962.
21. Williamson, L.M. and D.V. Devine, *Challenges in the management of the blood supply.* The Lancet, 2013. **381**(9880): p. 1866-1875.
22. Spahn, D.R. and L.T. Goodnough, *Alternatives to blood transfusion.* The Lancet, 2013. **381**(9880): p. 1855-1865.
23. Dixon, S., et al., *Economic analysis of the implementation of autologous transfusion technologies throughout England.* International journal of technology assessment in health care, 2005. **21**(2): p. 234-239.
24. NHS Supply Chain.*NHS Supply Chain Catalogue*.2015 [accessed:August 2016] Available from: https://my.supplychain.nhs.uk/catalogue
25. UK Cell Salvage Action Group, *ICS Technical Fact Sheet: Swab Washing*. 2015.
26. Committee, J.F.*British National Formulary*.2016 [accessed:August 2016] Available from: https://bnf.nice.org.uk/
27. Curtis L, *Unit Costs of Health and Social Care 2015*. 2015, University of Kent: PSSRU.
28. Qureshi, H., et al., *BCSH guideline for the use of anti-D immunoglobulin for the prevention of haemolytic disease of the fetus and newborn.* Transfusion Medicine, 2014. **24**(1): p. 8-20.
29. Department of Health.*NHS Reference Costs 2014/2015*.2015 [accessed:August 2016] Available from: https://www.gov.uk/government/uploads/system/uploads/attachment_data/file/477919/2014-15_Reference_costs_publication.pdf
30. NHS National Tariff Payment System.*2016/17 National Prices*.2016 [accessed:August 2016] Available from: www.gov.uk/government/publications/national-tariff-payment-system-2014-to-2015
31. Tinegate, H., et al., *Guideline on the investigation and management of acute transfusion reactions Prepared by the BCSH Blood Transfusion Task Force.* British journal of haematology, 2012. **159**(2): p. 143-153.

For peer review only - http://bmjopen.bmj.com/site/about/guidelines.xhtml

462 **TABLES**

463

464

465 **Table 1 Probabilities used in the model**

	Trial data	Probability	Distribution
**Cell salvage intended**			
Cell salvage intended → allocated treatment received (machine is on)	1432 / 1498	0.96	Beta
Allocated treatment received → salvaged blood returned	726/1432	0.51	Beta
Allocated treatment received → salvaged blood not returned	703/1432	0.49	Beta
Salvaged blood returned → donor blood transfusion given	22/726	0.03	Beta
Salvaged blood returned → donor blood transfusion not given	704 /726	0.97	Beta
Salvaged blood not returned → donor blood transfusion given	9/703	0.01	Beta
Salvaged blood not returned → donor blood transfusion not given	697 / 703	0.99	Beta
Cell salvage intended → allocated treatment not received (machine is off)	66/1498	0.04	Beta
Allocated treatment not received → donor blood transfusion given	6/66	0.09	Beta
Allocated treatment not received → donor blood transfusion not given	60/66	0.91	Beta
**Standard care intended**			
Standard care intended → allocated treatment received (machine is off)	1434/1492	0.96	Beta
Allocated treatment received → donor blood transfusion given	47/1434	0.03	Beta
Allocated treatment received → donor blood transfusion not given	1387 / 1434	0.97	Beta
Standard care intended → allocated treatment not received (machine is on)	58/1492	0.04	Beta
Allocated treatment not received → salvaged blood returned	35/58	0.60	Beta
Allocated treatment not received → salvaged blood not returned	23/58	0.40	Beta
Salvaged blood returned → donor blood transfusion given	4/35	0.11	Beta
Salvaged blood returned → donor blood transfusion not given	31/35	0.89	Beta
Salvaged blood not returned → donor blood transfusion given	1/23	0.04	Beta
Salvaged blood not returned → donor blood transfusion not given	22/23	0.96	Beta

466

467

For peer review only - http://bmjopen.bmj.com/site/about/guidelines.xhtml

468 **Table 2 Intraoperative resource use and costs**

Item	Resource Use: Cell salvage (n=1498)	Resource Use: Control (n=1492)	Unit Cost	Mean cost per procedure: Cell salvage (n-=1498)	Mean cost per procedure: Control (n=1492)	Assumption / Working	Source
Running costs	1432	58	£6.14	£6.14	£6.14	Based on annual maintenance costs for Haemonetics Cell Saver 5 (Haemonetics UK Ltd) machine and estimated annual usage	UHB, (Mr Scott Hancock, University Hospitals Birmingham, 2016, personal communication); NICE costing statement blood transfusion (Nov 2015) [14]
Collection Set	1	1	£41.71	£41.71	£41.71	Based on the assumption that one collection set is used per procedure	NHS Supply Chain Catalogue Autotransfusion reservoir 3 l [24]
Processing Pack	1	1	£77	£77	£77	Based on the assumption that one processing pack is used per procedure	NHS Supply Chain Catalogue Intraoperative autologous blood system cell saver 5+ bowl set 125ml (Haemonetics UK Ltd) [24]
Leukocyte depletion filter	782	25	n/a	n/a	n/a	Cost not included in the analysis as leukocyte depletion filter included in the collection set for Haemonetics Cell Saver 5 machine (Haemonetics UK Ltd)	NHS Supply Chain Catalogue Autotransfusion reservoir 3 litre[24]
Additional sucker	598	29	£15.41	£6.43	£7.70	Mean cost based on the number of additional suckers used in each treatment arm / total number of patients who received cell salvage	NHS Supply Chain Catalogue Aspiration & anticoagulation line Cell Saver. (Haemonetics UK Ltd) £308.02 for 20[24]
Swab washing	781	21	£0.80	£0.44	£0.29	Mean cost based on the number of times swabs were washed in each treatment group/total number of patients who received cell salvage	ICS Factsheet 1 Swab Washing March 2015 [25], based on the cost of 1l of sodium chloride 0.9%, BNF[26]
Staff			£0.72 (min)	£11.57	£12.03	Based on the staff type most frequently called into theatre solely for the purposes of cell salvage	Unit cost for hospital based nurse, band 5, PSSRU unit costs 2015 (costs include qualifications) [27]
Saline (l)	2	2	£0.80	£1.60	£1.60	Based on the assumption that 2 l of saline would be administered to all patients undergoing cell salvage prior to collection[14]	Based on the cost of 1l of 0.9% sodium chloride, BNF[26]

For peer review only - http://bmjopen.bmj.com/site/about/guidelines.xhtml

Heparin sodium (30,000 IU)	2	2	£10.60	£21.20	£21.20	Based on the assumption that 60,000 IU of heparin would be administered to all patients undergoing cell salvage prior to collection[14]	Based on the cost of 1-ml amp of heparin sodium 25,000 IU/ml and 1-ml amp of heparin sodium 5,000 IU/ml, BNF[26]
Anti-D (500 IU)	1	1	£33.75	£3.04	£3.04	Based on the assumption that all RhD-negative women delivering a RhD-positive baby receive at least 500 IU of anti-D[28]. Mean cost per procedure based on the probability of a woman requiring anti-D in each treatment arm (0.09)	Based on the cost of 500-unit vial of anti-D immunoglobulin, BNF[26]
Anti-D (1500 IU)	1	1	£58	£5.22	£5.22	Based on the assumption that women who receive cell salvage are offered 1500 IU of anti-D[28]. Mean cost per procedure based on the probability of a woman requiring anti-D in each treatment arm (0.09)	Based on the cost of 1,500-unit vial of anti-D immunoglobulin, BNF[26]
RBC transfusion (units)	3	3	First unit: £194 Subsequent units: £166	£520	£520	Based on the assumption that all units transfused in each treatment arm were RBC[14]	NICE costing statement for blood transfusion (November 2015)[14]. Unit cost for RBCs obtained from NHSBT 2016/17[15]

For peer review only

Table 3 Postoperative resource use and costs

	Resource Use			Mean cost per patient		
**Item**	**Cell salvage** (n=1498)	**Control** (n=1492)	**Unit Cost**	**Cell salvage** (n-=1498)	**Control** (n=1492)	**Source**
Inpatient stay (normal days)	3734.5	3852	£431.45	£1,074	£1,113	NHS reference costs 2014/15[29]. Weighted average unit cost for elective and non-elective inpatient bed days
Inpatient stay (HLC)	189.5	136	£539 - £848*	£78	£56	NHS reference costs 2014/15 [29] National tariff payment system 2016/17 [30]
Adverse events	3	0	n/a	n/a	n/a	Assumption that transfusion would be discontinued in the event of an adverse reaction based on BCSH guidelines [31]
Hospital transfer	2	2	£99	£0.13	£0.13	PSSRU 2015 [27]
Investigations	6	10	£94 - £138†	£0.42	£0.70	NHS reference costs 2014/15 [29]
Additional surgery	11	8	£399 - £2,991‡	£13	£9	NHS reference costs 2014/15 [29]
RBC transfusion (units)	3	3	First unit: £194 Subsequent units: £166	£13	£17	NICE costing statement for blood transfusion (November 2015). Unit cost for RBC obtained from NHSBT 2016/17 [15]
Total cost of postnatal care per patient				£1178.55	£1195.86	

* Range based on cost per day of care: Level 1 £539, Level 2 £674, Level 3 £848
† Range based on unit cost of a CT scan (£94) and an MRI scan (£138)
‡ Range based on unit cost of additional surgeries (less cost of days in hospital)

For peer review only - http://bmjopen.bmj.com/site/about/guidelines.xhtml

476 **Table 4 Results**

Transfusion strategy	Average cost per patient (£)	Difference in costs (£)	Effectiveness (Donor blood transfusion avoided)	Incremental donor blood transfusion avoided	ICER per donor blood transfusion avoided (£)
**Analysis ITT**					
Standard care	1244		0.965		
Cell salvage	1327	83	0.975	0.010	8110
**Analysis PP**					
Standard care	1238		0.967		
Cell salvage	1330	92	0.978	0.011	8252
**Analysis ECS**					
Standard care	1352		0.986		
Cell salvage	1407	55	0.990	0.004	13713

For peer review only - http://bmjopen.bmj.com/site/about/guidelines.xhtml

Table 5 Deterministic sensitivity analysis

	Original value		Revised value	Original result		
				Cost per donor blood transfusion avoided		
				**Analysis ITT**	**Analysis PP**	**Analysis ECS**
				£8110	£8252	£13,713
					**Revised result**	
**(i) Equipment and disposables required for the cell salvage procedure**						
Applying the cost of consumables (collection set + processing pack) used by NICE	£118.71		£119.75	£8205	£8346	£13,952
Including acquisition costs based on Haemonetics Cell Saver machine	-		£22.13	£10,114	£10,246	£18,805
	Cell salvage	Control				
Using a continuous transfusion cell saver where the machine is only set up for processing in patients having blood returned and swab washing is not conducted	£125.14	£126.40	£34.73	£1022	£1184	Dominates
**(ii) Staff**						
No additional member of staff being called into theatre solely for the purposes of cell salvage	Cell salvage £11.57	Control £12.03	£0	£7065	£7210	£10,932
**(iii) Donor blood**						
Variation in the estimate of the cost of a 3 unit RBC transfusion	£520		£750	£7886	£8028	£13,330
			£1000	£7636	£7778	£13,080
			£1250	£7386	£7528	£12,830
			£1500	£7136	£7278	£12,580
For routine cell salvage to be considered cost-effective in this model the price of a 3 unit RBC transfusion would have to be:				£8637	£8778	£13,186

For peer review only - http://bmjopen.bmj.com/site/about/guidelines.xhtml

For peer review only

FIGURES

Figure 1 Decision tree structure

Figure 2 Cost-effectiveness acceptability curve for donor blood transfusion avoided

For peer review only

For peer review only - http://bmjopen.bmj.com/site/about/guidelines.xhtml

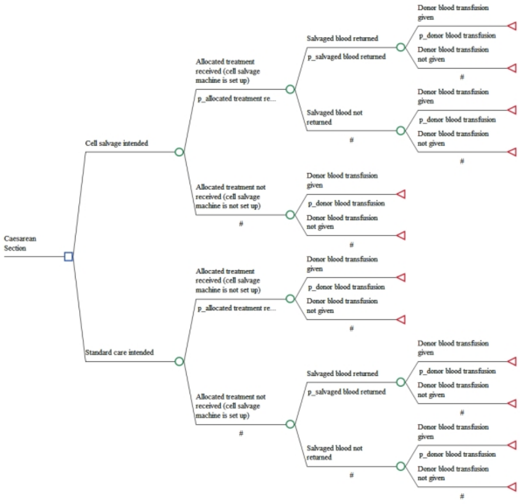

Decision tree structure

167x86mm (300 x 300 DPI)

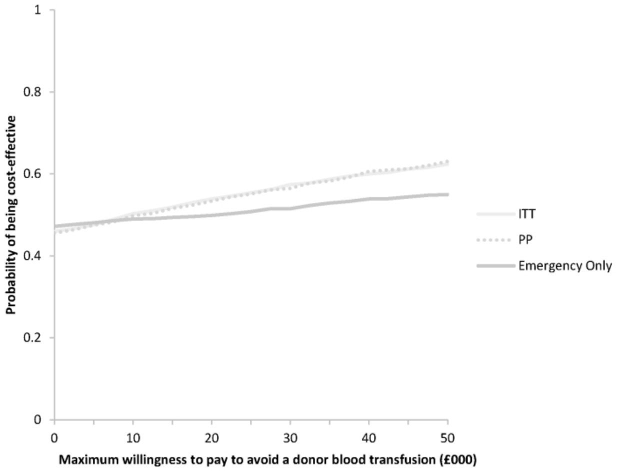

Cost-effectiveness acceptability curves for donor blood transfusion avoided

210x297mm (300 x 300 DPI)

The CHEERS Checklist is part of the CHEERS Statement. The CHEERS Statement has been endorsed and co-published by the following journals:

BJOG: An International Journal of Obstetrics and Gynaecology
BMC Medicine 2013; 11:80
BMJ 2013;346:f1049
Clinical Therapeutics 27 March 2013 (Article in Press DOI: 10.1016/j.clinthera.2013.03.003)
Cost Effectiveness and Resource Allocation 2013 11:6.
The European Journal of Health Economics 2013 Mar 26. [Epub ahead of print]
International Journal of Technology Assessment in Health Care
Journal of Medical Economics 2013 Mar 25. [Epub ahead of print]
Pharmacoeconomics 2013 Mar 26. [Epub ahead of print]
Value in Health 2013 March - April;16(2):e1-e5

CHEERS Checklist
Items to include when reporting economic evaluations of health interventions

Section/item	Item No	Recommendation	Reported on page No/ line No
**Title and abstract**			
Title	1	Identify the study as an economic evaluation or use more specific terms such as "cost-effectiveness analysis", and describe the interventions compared.	1/1
Abstract	2	Provide a structured summary of objectives, perspective, setting, methods (including study design and inputs), results (including base case and uncertainty analyses), and conclusions.	3/32
**Introduction**			
Background and objectives	3	Provide an explicit statement of the broader context for the study. Present the study question and its relevance for health policy or practice decisions.	6/92
**Methods**			
Target population and subgroups	4	Describe characteristics of the base case population and subgroups analysed, including why they were chosen.	7/118
Setting and location	5	State relevant aspects of the system(s) in which the decision(s) need(s) to be made.	7/118
Study perspective	6	Describe the perspective of the study and relate this to the costs being evaluated.	7/127
Comparators	7	Describe the interventions or strategies being compared and state why they were chosen.	7/121
Time horizon	8	State the time horizon(s) over which costs and consequences are being evaluated and say why appropriate.	7/130
Discount rate	9	Report the choice of discount rate(s) used for costs and	n/a

		outcomes and say why appropriate.	[illegible]
Choice of health outcomes	10	Describe what outcomes were used as the measure(s) of benefit in the evaluation and their relevance for the type of analysis performed.	7/128
Measurement of effectiveness	11a	*Single study-based estimates:* Describe fully the design features of the single effectiveness study and why the single study was a sufficient source of clinical effectiveness data.	n/a
	11b	*Synthesis-based estimates:* Describe fully the methods used for identification of included studies and synthesis of clinical effectiveness data.	n/a
Measurement and valuation of preference based outcomes	12	If applicable, describe the population and methods used to elicit preferences for outcomes.	n/a
Estimating resources and costs	13a	*Single study-based economic evaluation:* Describe approaches used to estimate resource use associated with the alternative interventions. Describe primary or secondary research methods for valuing each resource item in terms of its unit cost. Describe any adjustments made to approximate to opportunity costs.	n/a
	13b	*Model-based economic evaluation:* Describe approaches and data sources used to estimate resource use associated with model health states. Describe primary or secondary research methods for valuing each resource item in terms of its unit cost. Describe any adjustments made to approximate to opportunity costs.	7/133
Currency, price date, and conversion	14	Report the dates of the estimated resource quantities and unit costs. Describe methods for adjusting estimated unit costs to the year of reported costs if necessary. Describe methods for converting costs into a common currency base and the exchange rate.	8/143
Choice of model	15	Describe and give reasons for the specific type of decision-analytical model used. Providing a figure to show model structure is strongly recommended.	7/133
Assumptions	16	Describe all structural or other assumptions underpinning the decision-analytical model.	7/133
Analytical methods	17	Describe all analytical methods supporting the evaluation. This could include methods for dealing with skewed, missing, or censored data; extrapolation methods; methods for pooling data; approaches to validate or make adjustments (such as half cycle corrections) to a model; and methods for handling population heterogeneity and uncertainty.	9/164
**Results**			
Study parameters	18	Report the values, ranges, references, and, if used, probability distributions for all parameters. Report reasons or sources for distributions used to represent uncertainty where appropriate. Providing a table to show the input values is strongly recommended.	7/138

Incremental costs and outcomes	19	For each intervention, report mean values for the main categories of estimated costs and outcomes of interest, as well as mean differences between the comparator groups. If applicable, report incremental cost-effectiveness ratios.	10/202
Characterising uncertainty	20a	*Single study-based economic evaluation:* Describe the effects of sampling uncertainty for the estimated incremental cost and incremental effectiveness parameters, together with the impact of methodological assumptions (such as discount rate, study perspective).	n/a
	20b	*Model-based economic evaluation:* Describe the effects on the results of uncertainty for all input parameters, and uncertainty related to the structure of the model and assumptions.	11/224
Characterising heterogeneity	21	If applicable, report differences in costs, outcomes, or cost-effectiveness that can be explained by variations between subgroups of patients with different baseline characteristics or other observed variability in effects that are not reducible by more information.	n/a
**Discussion**			
Study findings, limitations, generalisability, and current knowledge	22	Summarise key study findings and describe how they support the conclusions reached. Discuss limitations and the generalisability of the findings and how the findings fit with current knowledge.	13/264
**Other**			
Source of funding	23	Describe how the study was funded and the role of the funder in the identification, design, conduct, and reporting of the analysis. Describe other non-monetary sources of support.	4/76
Conflicts of interest	24	Describe any potential for conflict of interest of study contributors in accordance with journal policy. In the absence of a journal policy, we recommend authors comply with International Committee of Medical Journal Editors recommendations.	5/83

For consistency, the CHEERS Statement checklist format is based on the format of the CONSORT statement checklist

The CHEERS Statement may be accessed by the publication links above.

The **ISPOR CHEERS Task Force Report** provides examples and further discussion of the 24-item CHEERS Checklist and the CHEERS Statement. It may be accessed via the *Value in Health* link or via the ISPOR Health Economic Evaluation Publication Guidelines – CHEERS: Good Reporting Practices webpage: http://www.ispor.org/TaskForces/EconomicPubGuidelines.asp

The citation for the CHEERS Task Force Report is:
Husereau D, Drummond M, Petrou S, et al. Consolidated health economic evaluation reporting standards (CHEERS)—Explanation and elaboration: A report of the ISPOR health economic evaluations publication guidelines good reporting practices task force. Value Health 2013;16:231-50.
